# Ca^2+^ entry into neurons is facilitated by cooperative gating of clustered Ca_V_1.3 channels

**DOI:** 10.7554/eLife.15744

**Published:** 2016-05-17

**Authors:** Claudia M Moreno, Rose E Dixon, Sendoa Tajada, Can Yuan, Ximena Opitz-Araya, Marc D Binder, Luis F Santana

**Affiliations:** 1 Department of Physiology and Membrane Biology, University of California, Davis, United States; 2 Department of Physiology and Biophysics, University of Washington, Seattle, United States; The University of Texas at Austin, United States

**Keywords:** CaV1.3 channels, hippocampal neurons, calcium facilitation, Rat

## Abstract

Ca_V_1.3 channels regulate excitability in many neurons. As is the case for all voltage-gated channels, it is widely assumed that individual Ca_V_1.3 channels behave independently with respect to voltage-activation, open probability, and facilitation. Here, we report the results of super-resolution imaging, optogenetic, and electrophysiological measurements that refute this long-held view. We found that the short channel isoform (Ca_V_1.3_S_), but not the long (Ca_V_1.3_L_), associates in functional clusters of two or more channels that open cooperatively, facilitating Ca^2+^ influx. Ca_V_1.3_S_ channels are coupled via a C-terminus-to-C-terminus interaction that requires binding of the incoming Ca^2+^ to calmodulin (CaM) and subsequent binding of CaM to the pre-IQ domain of the channels. Physically-coupled channels facilitate Ca^2+^ currents as a consequence of their higher open probabilities, leading to increased firing rates in rat hippocampal neurons. We propose that cooperative gating of Ca_V_1.3_S_ channels represents a mechanism for the regulation of Ca^2+^ signaling and electrical activity.

**DOI:** http://dx.doi.org/10.7554/eLife.15744.001

## eLife digest

The electrical charge inside a cell is different from that outside of the cell. Neurons rely on this difference to send signals via electrical impulses. This process involves ions moving across the neuron’s membrane through proteins called ion channels. Ca_v_1.3 channels are ion channels that open when the membrane’s electrical charge changes to allow positively charged calcium ions into the cell. This generates an electrical current that enables neurons in the brain to produce repetitive impulses.

Calcium ions entering through a Ca_v_1.3 channel can encourage the channel to allow in even more calcium ions. A closely related channel called Ca_v_1.2, which is essential to the activity of heart muscle, behaves in a similar way. Researchers have recently found that Ca_v_1.2 channels are arranged in clusters in the membrane and that adjacent channels interact to allow more calcium ions through the channels. This was an unexpected finding because it had long been thought that all ion channels acted independently.

Moreno et al. have now used a range of different approaches to investigate the behavior of one form of the Ca_v_1.3 channel, called Ca_V_1.3_S_, in human cells and in neurons from rat brains. Initial experiments confirmed that calcium ions stimulated these channels to open in a coordinated way and to allow in more calcium. High-resolution microscopy then revealed that the Ca_V_1.3_S_ channels do form clusters in the cell membrane.

Moreno et al. went on to demonstrate that this simultaneous opening of Ca_V_1.3_S_ channels first requires a protein called calmodulin to bind calcium inside the cell. Next, the calcium-calmodulin complex associates with the parts of the channels that are also inside the cell. Further experiments showed that coupling the Ca_v_1.3 channels together allows them to cooperate, and makes them more likely to be open and generate bigger calcium flows, and allowed neurons to send electrical signals more frequently. Future challenges include investigating how the clusters of Ca_v_1.3 channels are established and maintained, and determining how the channels’ cooperation plays a role in both healthy and diseased states.

**DOI:** http://dx.doi.org/10.7554/eLife.15744.002

## Introduction

Ca_V_1.3 channels are widely expressed in neurons throughout the brain and spinal cord (Tan et al., 2011), where they serve a number of critical functions including the modulation of resting potentials, the amplification of synaptic currents and the generation and shaping of repetitive firing (Guzman et al., 2009; Olson et al., 2005; Striessnig et al., 2006). These channels are dihydropyridine-sensitive L-type Ca^2+^ channels composed of a pore-forming Ca_V_1.3α_1_ subunit and accessory β and α_2_-δ subunits. The carboxy-terminus (C-terminus) of the α_1D_ subunit is structurally complex, containing an EF hand domain as well as pre-IQ and IQ domains to which the Ca^2+^-binding protein calmodulin (CaM) binds (Ben-Johny and Yue, 2014).

**Figure 1.**
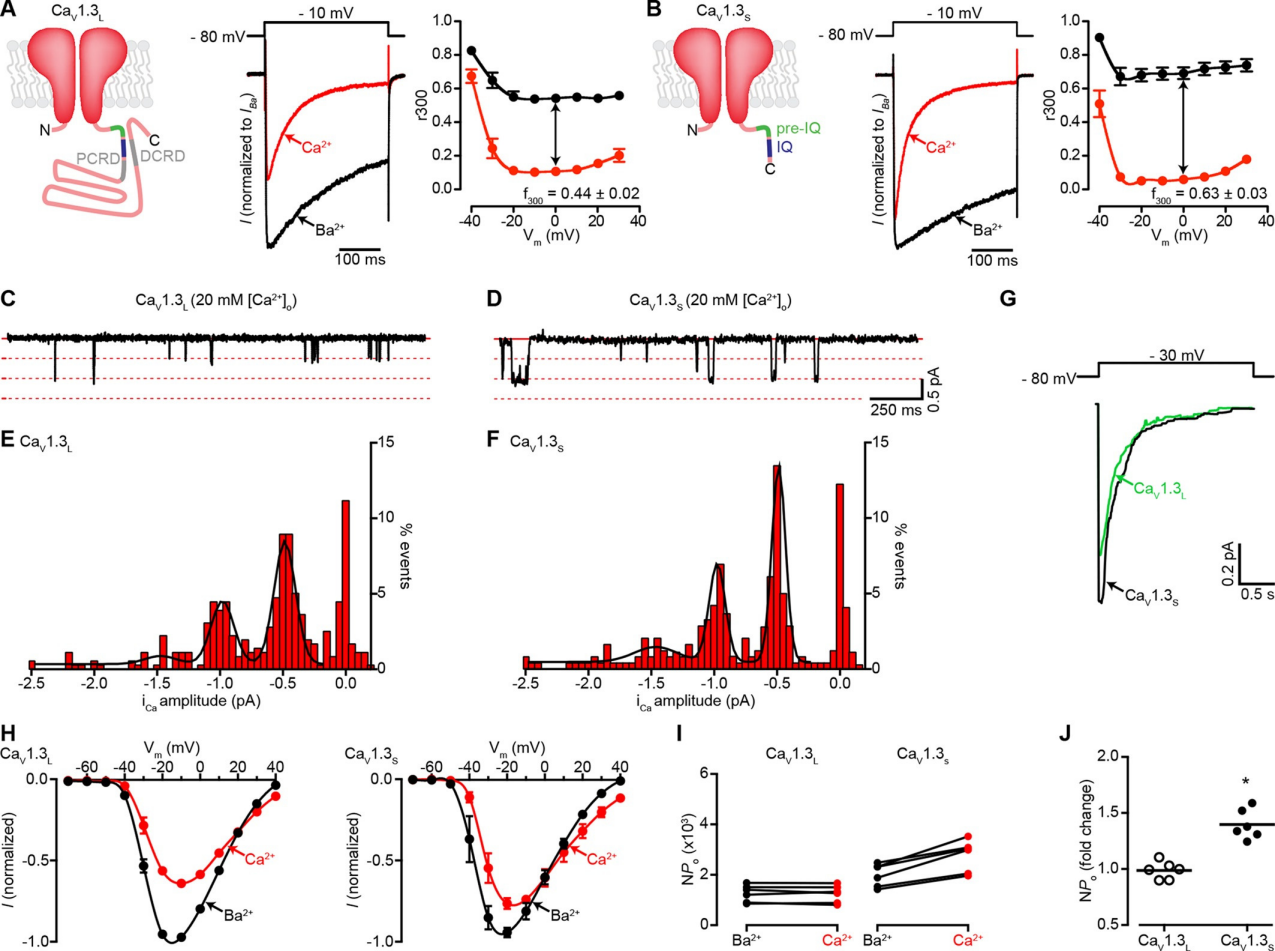
Ca^2+^ enhances the activity of Ca_V_1.3_S_, but not Ca_V_1.3_L_, channels. (**A**) *Left*: Schematic of Ca_V_1.3_L_ channel splice variant, depicting the domains important for Ca^2+^-mediated regulation: pre-IQ (green), IQ (blue), proximal and distal C-terminal regulatory domains (PCRD, DCRD, gray). *Middle*: Representative *I_Ca_* and *I_Ba_* of Ca_V_1.3_L_ channels expressed in tsA-201 cells. Currents were evoked by a 300-ms depolarization from holding potential of -80 mV to a test potential of -10 mV, with 2 mM Ba^2+^ (black) or 2 mM Ca^2+^ (red) as the charge carrier in the same cell. *Right*: Voltage dependence of Ca_V_1.3_L_ channel CDI. r300 is the fraction of *I_Ca_* or *I_Ba_* remaining after 300 ms. f_300_ is the difference between *I_Ca_* and *I_Ba_* r300 values at 0 mV. (**B**) *Left*: Schematic of Ca_V_1.3_S_ channel splice variant. *Middle:* Representative *I_Ca_* and *I_Ba_* of Ca_V_1.3_S_ channels. Right: Voltage dependence of Ca_V_1.3_S_ channel CDI, format as in (**A**). *I_Ca_* is presented as normalized to *I_Ba_*, currents analyzed for these experiments were in a range between 200 and 900 pA. (**C** and **D**) Representative *i*_Ca_ single channel recordings from Ca_V_1.3_L_ (**C**) and Ca_V_1.3_S_ channels (**D**) expressed in tsA-201 cells during step depolarizations from -80 to -30 mV. (**E** and **F**) all-points *i*_Ca_ amplitude histograms for Ca_V_1.3_L _(**E**) and Ca_V_1.3_S_ channels (**F**), the black line is the best fit to the data with a multi-Gaussian function with a quantal unit value of -0.48 ± 0.07 for Ca_V_1.3_L_ and -0.49 ± 0.01 pA for Ca_V_1.3_S_ channels, respectively (constructed from *n* = 6 cells each). Single channel recordings were also performed using Ba^2+^ as the charge carrier for both channels (see Figure 1—figure supplement 1). (**G**) Ensemble average single-channel currents from multiple sweeps. (**H**) Current-voltage relationships of Ca_V_1.3_L_ currents (left) and Ca_V_1.3_S_ currents (right) in the presence of 2 mM Ca^2+^ (red) or Ba^2+^ (black) as the charge carrier. Data were normalized to the maximum current in the presence of Ba^2+^. Symbols are averages of 7 cells ± SEM. (**I**) Scatter plots of NP_o_ (at -10 mV) of Ca_V_1.3_L_ (left) and Ca_V_1.3_S_ (right) channels in the presence of Ba^2+^ and Ca^2+^. (**J**) Change in N*P_o_* for Ca_V_1.3_L_ and Ca_V_1.3_S_ channels for currents recorded in the presence of Ca^2+^ and then Ba^2+^. The horizontal bar shows the mean value (*p < 0.001). **DOI:** http://dx.doi.org/10.7554/eLife.15744.003

Alternative splicing results in the expression of 'long' and 'short' Ca_V_1.3 channel isoforms that differ in the length of the C-terminus (Singh et al., 2008). The splice variant 42A (Ca_V_1.3_S_) has a short C-terminus of 183 amino acids long compared to the 695 amino acids of the long isoform (Ca_V_1.3_L_). The Ca_V_1.3_S_ channels lack the so-called C-terminal modulatory domain (CTM), comprised of proximal (PCRD) and distal (DCRD) regulatory domains that block CaM binding to the IQ domain (Figures 1A and B, left). As a consequence, Ca_V_1.3_S_ channels activate at lower voltages, have a higher open probability, and inactivate faster than Ca_V_1.3_L_ channels (Bock et al., 2011; Singh et al., 2008; Tan et al., 2011).

**Figure 1—figure supplement 1.**
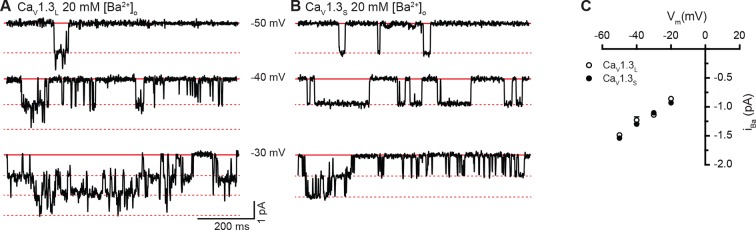
Single-channel recordings of *i_Ba_* for Ca_V_1.3_S_ and Ca_V_1.3_L_ channels. (**A** and **B**) Representative *i*_Ba_ single channel recordings from Ca_V_1.3_S _(**A**) and Ca_V_1.3_L_ channels (**B**) expressed in tsA-201 cells during step depolarizations from -80 to -30 mV. (**C**) Average single-channel current-voltage relationship ± SEM. Data from 5 cells for Ca_V_1.3_S_ and 7 cells for Ca_V_1.3_L_. **DOI:** http://dx.doi.org/10.7554/eLife.15744.004

Ca_V_1.3 channels carry about 20% of the L-type calcium current in hippocampal neurons (Moosmang et al., 2005). As Ca_V_1.3_S_ channels activate at low voltages, they are a particular good candidate for underlying the sustained the neuronal firing and the persistent low- voltage-activated current observed in CA3 neurons (Avery and Johnston, 1996). In fact, Ca_V_1.3 channels support spontaneous firing of dopaminergic neurons in the substantia nigra and mid-spiny striatal neurons (Guzman et al., 2009; Olson et al., 2005).

The function of Ca_V_1.3 channels is tightly regulated by changes in intracellular Ca^2+^ ([Ca^2+^]_i_). The opening of Ca_V_1.3 channels causes a local increase in [Ca^2+^]_i_ that induces two opposing regulatory mechanisms: Ca^2+^-dependent inactivation (CDI) and Ca^2+^-dependent facilitation (CDF) (Ben-Johny and Yue, 2014). CDF manifests as an increase in the magnitude of the Ca_V_1.3 current (*I_Ca_*) with repetitive activation. In neurons, CDF can induce a persistent *I_Ca_* that increases firing rate and may even lead to self-sustained firing (Fransen et al., 2006; Major and Tank, 2004; Sheffield et al., 2013). It has been proposed that CDF of the Ca_V_1.3 channel depends on Ca^2+^/CaM-dependent kinase II (CaMKII)-mediated phosphorylation, as has also been proposed for the closely related Ca_V_1.2 channel (Hudmon et al., 2005; Xiao et al., 1994; Yuan and Bers, 1994). This phosphorylation requires the presence of a second protein, densin, which binds to the PDZ domain located in the most distal part of the C-terminus of the channel (Jenkins et al., 2010). Because Ca_V_1.3_S_ lacks that PDZ domain, CaMKII-mediated phosphorylation is unlikely to be responsible for CDF in Ca_V_1.3_S_ channels. Thus, the mechanisms underlying the CDF of the widely expressed Ca_V_1.3_S_ channel have not yet been resolved.

Two recent studies by Dixon et al. (Dixon et al., 2012; 2015) have suggested the tantalizing hypothesis that Ca^2+^-induced interactions between the C-termini of neighboring Ca_V_1.2 channels facilitates Ca^2+^ influx by increasing the activity of adjoined channels in cardiac muscle. At present, however, whether this physical and functional coupling of Ca_V_1.2 channels is a common mechanism for the control of Ca^2+^ influx via voltage-gated Ca^2+^ channel function, including Ca_V_1.3 channels is unknown. Furthermore, the possibility that cooperative Ca_V_1.3 channel gating regulates neuronal excitability is also unclear.

In the present study, using electrophysiological, optogenetic, and super-resolution imaging approaches, we discovered that Ca_V_1.3_S_ channels form functional clusters of two or more channels along the surface membrane of hippocampal neurons. Clustered Ca_V_1.3_S_ channels undergo Ca^2+^-induced physical interactions that increase the activity of adjoined channels, facilitate Ca^2+^ currents and thereby increase firing rates in hippocampal neurons. We propose that cooperative gating of Ca_V_1.3_S_ channels is a new general mechanism for the amplification of Ca^2+^ signals in excitable cells.

## Results

### A Ca^2+^-dependent mechanism mediates facilitation of Ca_V_1.3_S_, but not Ca_V_1.3_L_, channels

Because Ca_V_1.3 channels are alternatively spliced, we first sought to determine whether Ca_V_1.3_S_ and Ca_V_1.3_L_ channels are differentially regulated by [Ca^2+^]_i_. Macroscopic currents were recorded from tsA-201 cells expressing either Ca_V_1.3_S_ or Ca_V_1.3_L_ channels in the presence of 2 mM Ba^2+^ or 2 mM Ca^2+^. Currents were activated by a depolarizing pulse (300 ms) from a holding potential of -80 mV to -10 mV. With Ba^2+^ in the external solution, membrane depolarization induced large Ca_V_1.3_L_ currents that inactivated slowly (Figure 1A, center). Switching to a perfusion solution containing Ca^2+^ decreased the amplitude of Ca_V_1.3_L_ currents by nearly 40% and increased the rate of inactivation. Like Ca_V_1.3_L_ currents, Ca_V_1.3_S_ currents inactivated faster when Ca^2+^ was used as a charge carrier however, in agreement with previous reports (Bock et al., 2011; Singh et al., 2008), we observed more pronounced CDI (defined as the difference between inactivation of *I_Ba_* and *I_Ca_*) in Ca_V_1.3_S_ channels compared to the Ca_V_1.3_L_ variant (Figure 1B, right versus Figure 1A, right). As discussed above, this difference in the magnitude of CDI has been attributed to the lack of the CTM domain in Ca_V_1.3_S_ channels. Curiously, the amplitude of Ca_V_1.3_S_ currents decreased to a lesser extent (only about 15% at -10 mV) upon changing the external solution from Ba^2+^ to Ca^2+^ (Figure 1B, center).

We investigated whether differences in the amplitude of elementary Ca_V_1.3_L_ and Ca_V_1.3_S_ channel currents could, at least in part, account for these disparities in macroscopic Ca^2+^ and Ba^2+^ currents. Single Ca_V_1.3_L_ and Ca_V_1.3_S_ channel currents were recorded from cell-attached patches with pipettes containing 20 mM Ca^2+^ or Ba^2+^. With Ca^2+^ as the charge carrier, the amplitudes of elementary Ca_V_1.3_S_ and Ca_V_1.3_L_ channel currents were similar. For example, at -30 mV they were -0.48 ± 0.07 and -0.49 ± 0.01 pA for Ca_V_1.3_L_ and Ca_V_1.3_S_ channels, respectively (Figures 1C–F). These values are in accordance with the unitary currents reported by Guia et al. for cardiac L-type channels using Ca^2+^ as charge carrier (Guia et al., 2001). Ensemble averages revealed currents that activated quickly and then inactivated likely due to Ca^2+^ and voltage-dependent mechanisms (Figure 1G). Furthermore, as is the case for other Ca_V_1 channels, the single channel currents produced during the opening of Ca_V_1.3_L_ and Ca_V_1.3_S_ channels were both larger with Ba^2+^ as the charge carrier than with Ca^2+^ (-1.14 ± 0.02 pA and -1.10 ± 0.02 pA for Ca_V_1.3_L_ and Ca_V_1.3_S_ at -30 mV, respectively), but not significantly different from each other (Figure 1—figure supplement 1). The amplitude of the unitary currents with Ba^2+^ is also in accordance with previously reported values for these channels (Bock et al., 2011). These data suggest that the difference observed in the macroscopic currents between the Ca_V_1.3_L_ and Ca_V_1.3_S_ channels is not due to differences in unitary currents.

We then tested the hypothesis that Ca^2+^ enhances the activation of Ca_V_1.3_S_, but not Ca_V_1.3_L_ channels by increasing the channel activity (*NP*_o_). We performed a quantitative analysis of *NP*_o_. The whole-cell current (I) is given by the equation I = i*N*P_o_, where *i* is the amplitude of the elementary current, *N* is the number of functional channels, and *P*_o_ is the channel open probability. Since elementary Ca_V_1.3_L_ and Ca_V_1.3_S_ currents are larger when Ba^2+^ rather than Ca^2+^ is the charge carrier, for I_Ca_ to be similar to I_Ba_, the *NP*_o_ of Ca_V_1.3_S_ must be higher in the presence of Ca^2+^ than Ba^2+^. We estimated *NP*_o_ with Ca^2+^ and Ba^2+^ as charge carriers by dividing the amplitude of whole-cell Ca_V_1.3_S_ and Ca_V_1.3_L_ currents at -10 mV, by the values of unitary current. Our analysis relies on the assumption that the relatively larger peak of Ca_V_1.3_S_ currents in the presence of Ca^2+^ was not due to faster activation kinetic of this channel than that of Ca_V_1.3_L_ channels. This assumption is reasonable, as we found no significant difference in the activation time constants for Ca_V_1.3_L_ and Ca_V_1.3_S_ currents that were 1.60 ± 0.22 and 1.17 ± 0.051 ms (-10 mV, n = 6 for each channel, p = 0.112), respectively. We used elementary currents values recorded with 2 mM Ca^2+^ (-0.16 pA) and Ba^2+^ (-0.24 pA) at -10 mV (Guia et al., 2001). We found that Ca^2+^ ions entering the cell through the channels increased the *NP*_o_ nearly 1.5-fold for Ca_V_1.3_S_ channels, but not at all for Ca_V_1.3_L_ channels (Figures 1I and J). Thus, assuming that the number of functional Ca_V_1.3 channels (*N*) in the membrane remained constant, a reasonable assumption given the short time lapse (~2 min) between recording I_Ba_ and I_Ca_ from the same cell, these data suggest that a Ca^2+^-dependent mechanism enhances inward Ca^2+^ currents by increasing the *P*_o_ of Ca_V_1.3_S_ channels, but not that of Ca_V_1.3_L_ channels.

### Ca_V_1.3_S_ channels gate cooperatively, increasing Ca^2+^ influx

**Figure 2.**
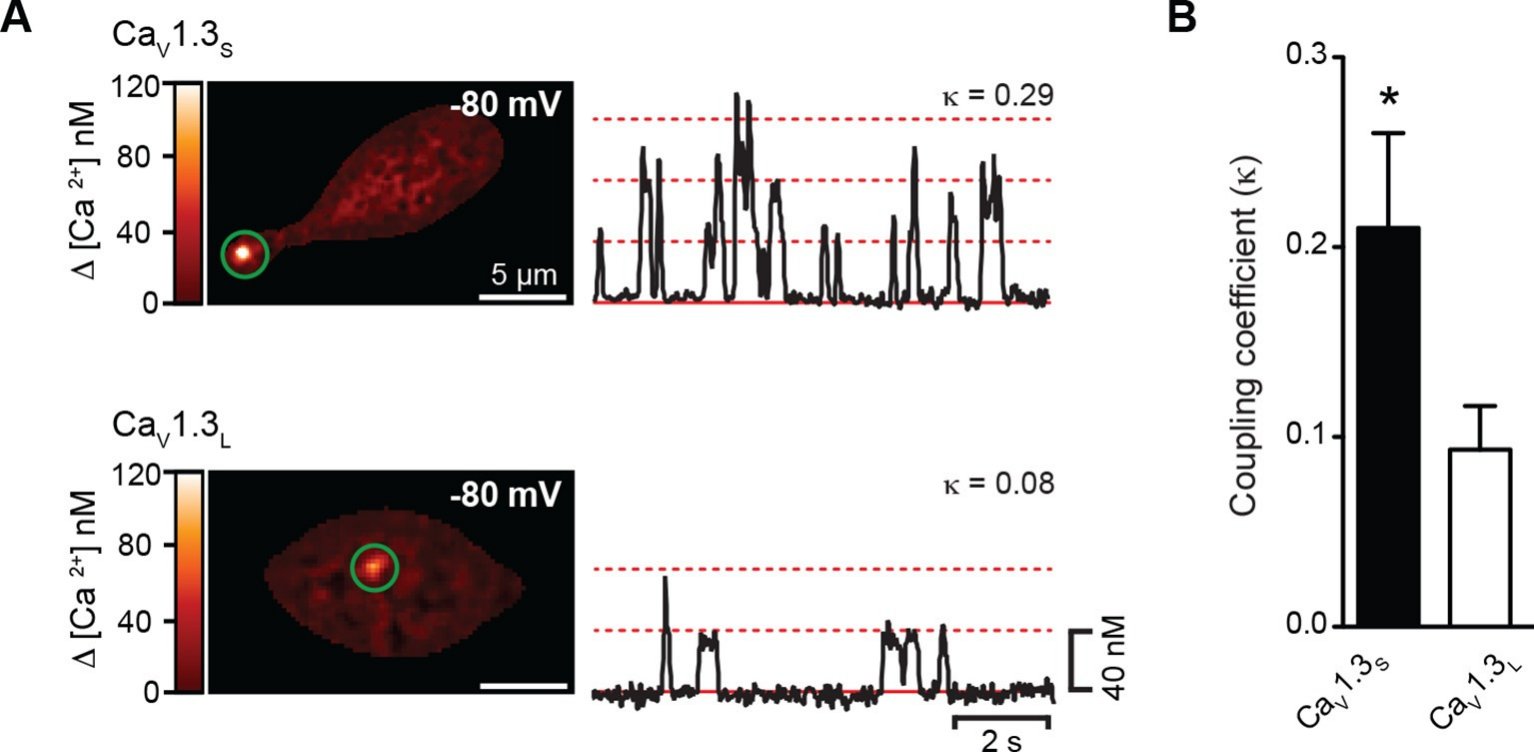
Ca_V_1.3_S_ but not Ca_V_1.3_L_ channels gate cooperatively to increase Ca^2+^ influx. (**A**) TIRF images of spontaneous Ca_V_1.3_S_ (top) and Ca_V_1.3_L_ sparklets (bottom) at a holding potential of -80 mV in tsA-201 cells expressing the respective channels. Traces at the right show the time course of [Ca^2+^]_i_ in the sites marked by the green circles. The dotted red lines show the amplitudes of 1 to 3 quantal levels. The coupling coefficient (κ) is shown above each trace. (**B**) Bar chart showing the coupling coefficient for the Ca_V_1.3_S_ and Ca_V_1.3_L_ sparklets sites. Bars are averages of 5 cells ± SEM (*p<0.05). **DOI:** http://dx.doi.org/10.7554/eLife.15744.005

One possible explanation for the Ca^2+^-influx–dependent increase in the *P*_o_ of Ca_V_1.3_S_ channels is cooperative gating among channels in small clusters, as we have previously reported for Ca_V_1.2 channels in cardiomyocytes and smooth muscle cells (Navedo et al., 2010). To test this possibility, we made optical recordings of individual Ca_V_1.3_S_-mediated Ca^2+^ influx events (called 'sparklets') in Ca_V_1.3_L_ and Ca_V_1.3_S_-expressing tsA-201 cells loaded with 200 µM Rhod-2 using total internal reflection fluorescence (TIRF) microscopy. Ca_V_1.3_S_ sparklets were recorded at a membrane potential of -80 mV in the presence of 20 mM external Ca^2+^; 10 mM EGTA was included in the patch pipette to confine the [Ca^2+^]_i_ signal to within ~1 µm of the point of Ca^2+^ entry (Zenisek et al., 2003). A quantal analysis of Ca_V_1.3_S_ and Ca_V_1.3_L_ sparklets revealed the presence of single-level (elementary) events with a mean amplitude of ~40 nM for both channels, in agreement with our previous study (Navedo et al., 2007). Interestingly, consistent with our single channel data, we found that multi-quantal sparklets, which presumably result from the simultaneous opening of several Ca_V_1.3 channels, were more commonly observed in cells expressing Ca_V_1.3_S_ than Ca_V_1.3_L_ channels (Figure 2A). To determine whether channels in a cluster opened cooperatively or independently, we calculated the coupling coefficient (κ) among channels within a Ca_V_1.3_S_ and Ca_V_1.3_L_ sparklet site by applying a coupled Markov-chain model (Chung and Kennedy, 1996). Channels with κ > 0.1 were considered coupled (Navedo et al., 2010). Using this approach, we found that the average κ value for Ca_V_1.3_S_ and Ca_V_1.3_L_ channels was 0.21 ± 0.05 (n = 15) and 0.08 ± 0.04 (n = 12), respectively (Figure 2B). These results support the hypothesis that Ca_V_1.3_S_ channels are more likely to undergo cooperative gating, generating persistent and greater Ca^2+^ influx than Ca_V_1.3_L_ channels.

### Ca_V_1.3 channels in hippocampal neurons aggregate in dense clusters

**Figure 3.**
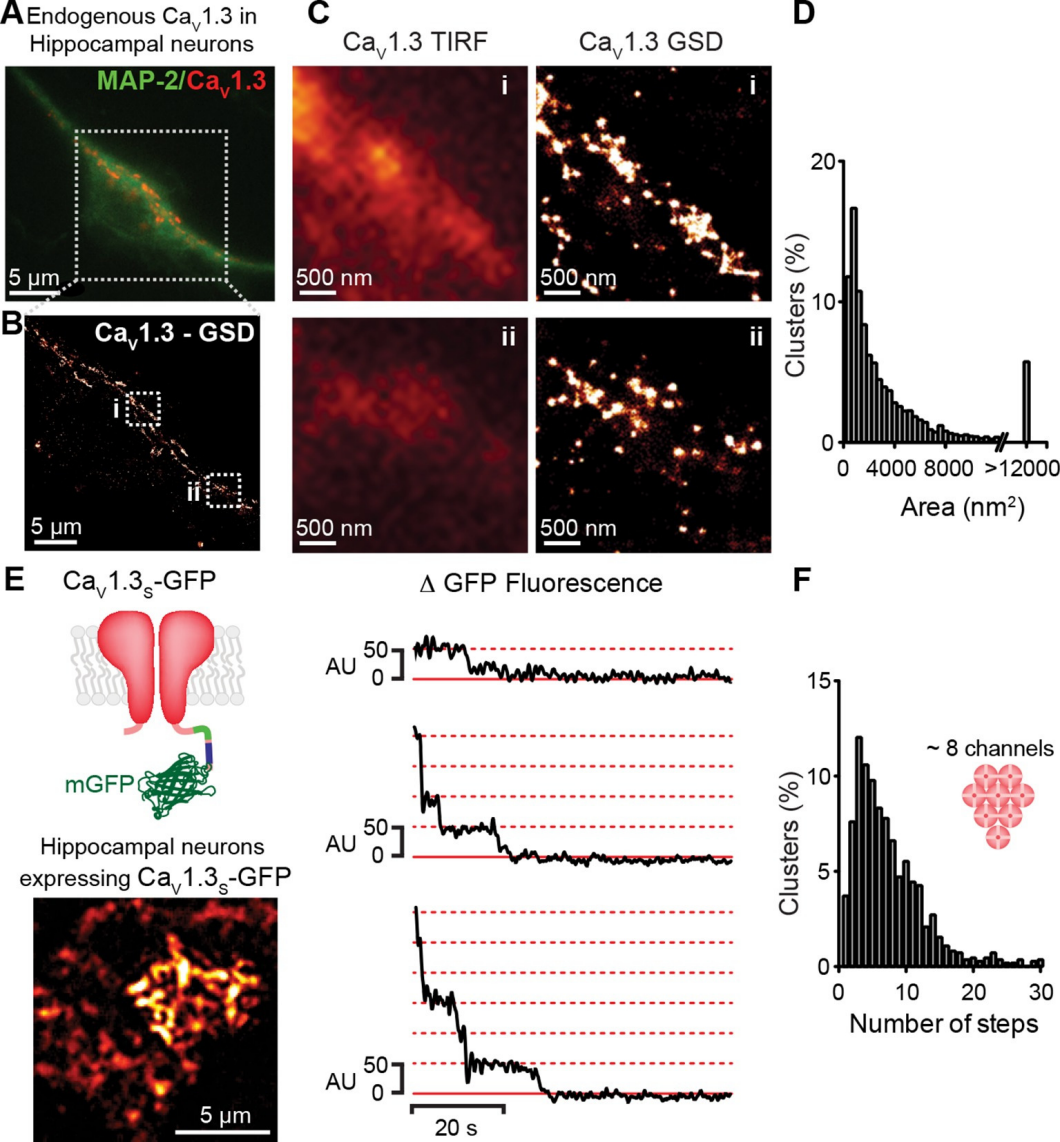
Ca_V_1.3 channels assemble into clusters in the plasma membrane of cultured hippocampal neurons. (**A**) Wide-field image of a representative cultured hippocampal neuron immunostained for Ca_V_1.3 channels (red) and the neuronal marker microtubule-associated protein 2 (MAP2; green). (**B**) Super-resolution (GSD) image of Ca_V_1.3 channels in the outlined region in (**A**). (**C**) Comparison of conventional (TIRF, *left*) and super-resolution (GSD, *right*) images of Ca_V_1.3 clusters in zones i and ii outlined in (**B**). (**D**) Frequency distribution of the area of Ca_V_1.3 channel clusters (n = 5320 clusters from 5 cells). (**E**) TIRF image of Ca_V_1.3_S_-mGFP channels expressed in cultured hippocampal neurons (*left*). Examples of sequential photobleaching steps for three different clusters (*right*). (**F**) Frequency distribution of Ca_V_1.3_S_ cluster bleaching steps (n = 1105 clusters from 18 cells). Clustering of Ca_V_1.3_S_ and Ca_V_1.3_L_ channels was tested in tsA-201 cells expressing the respective isoform (See also Figure 3—figure supplement 1). **DOI:** http://dx.doi.org/10.7554/eLife.15744.006

If the signal for cooperative Ca_V_1.3_S_ channel gating is a local increase in [Ca^2+^]_i_, these channels must be in close proximity to one another. To test this hypothesis, we examined the spatial organization of endogenous Ca_V_1.3 channels in hippocampal neurons using super-resolution localization microscopy (Figure 3A–D). Hippocampal neurons were immunostained against Ca_V_1.3 using an antibody kindly provided by Dr. William Catterall and Dr. Ruth Westenbroek. This analysis showed that Ca_V_1.3 channels form clusters occupying an average area of 3660 ± 80 nm^2^ (n = 5). The antibody used in this study has been shown to not recognize the corresponding sequence of the closely related Ca_V_1.2 α subunit in both, transfected cells and hippocampal tissue (Hell et al., 1993; 2013). However, we were not able to test the specificity of the antibody on Ca_V_1.3-KO neurons and thus, pursued our analyses of channel clustering using a heterologous expression of Ca_V_1.3 channels in tsA-201 cells.

**Figure 3—figure supplement 1.**
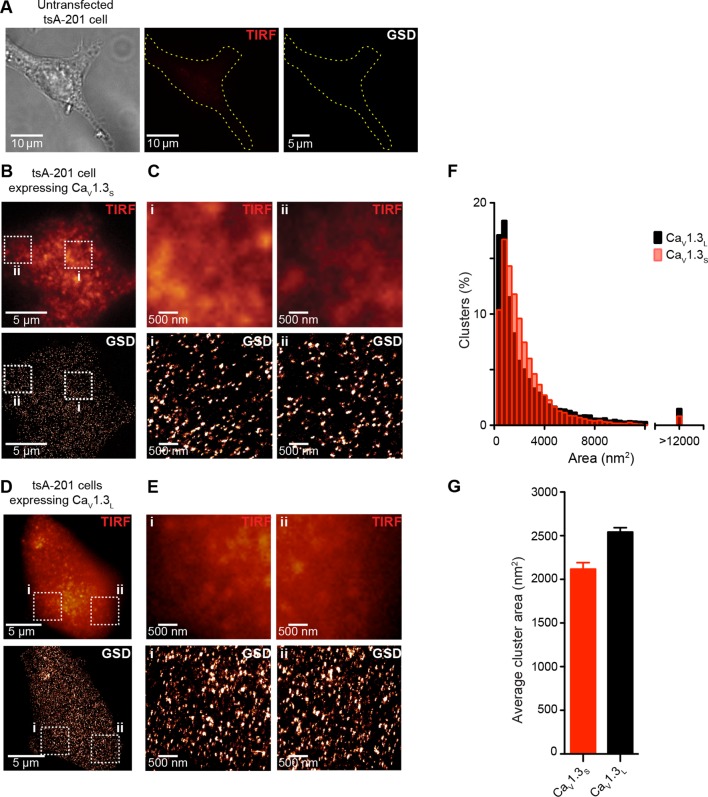
Ca_V_1.3_S_ and Ca_V_1.3_L_ channels form clusters in tsA-201 cells. (**A**) Untransfected tsA-201 cells immunostained against Ca_V_1.3 channels. Bright field (*left*), TIRF (*center*) and super-resolution (GSD, *right*) images, outline depicts the periphery of the cell. (**B**) TIRF (*top*) and GSD (*bottom*) of the immunostaining of a representative tsA-201 cell expressing Ca_V_1.3_S_ channels. (**C**) Zoom in of the Ca_V_1.3_S_ clusters in zones i and ii outlined in (**B**). (**D**) TIRF (*top*) and GSD images (*bottom*) of the immunostaining of a representative tsA-201 cell expressing Ca_V_1.3_L_ (**E**) Zoom in of Ca_V_1.3_L_ clusters in zones i and ii in (**D**). (**F**) Frequency distributions of the area of Ca_V_1.3_S_ and Ca_V_1.3_L_ channel clusters (n = 19,143 clusters from 7 cells for Ca_V_1.3_S_ and 15,580 cluster from 5 cells for Ca_V_1.3_L_). (**G**) Bar plot of the average cluster area for Ca_V_1.3_S_ and Ca_V_1.3_L_ channel clusters (n = 7 and 5 cells). **DOI:** http://dx.doi.org/10.7554/eLife.15744.007

**Figure 3—figure supplement 2.**
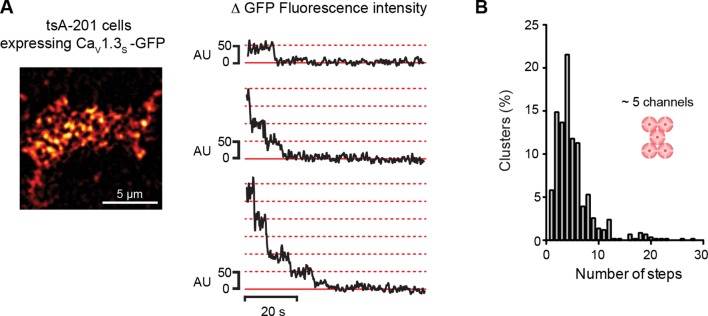
Ca_V_1.3_S_ organize in clusters of ~5 channels in tsA-201 cells. (**A**) TIRF image of Ca_V_1.3_S_-mGFP channels expressed in tsA-201 cells (*left*). Sequential photobleaching steps for three different clusters (*right*). (**B**) Frequency distribution of Ca_V_1.3_S_ cluster bleaching steps (n = 585 clusters from 11 cells). **DOI:** http://dx.doi.org/10.7554/eLife.15744.008

The specificity of the antibody in tsA-201 cells was tested by immunostaining untransfected and Ca_V_1.3_S_-transfected cells. No evidence of staining was observed in the untransfected cells (Figure 3—figure supplement 1A, n = 4). As our antibody cannot distinguish between Ca_V_1.3 channels isoforms, we expressed Ca_V_1.3_L_ or Ca_V_1.3_S_ channels separately and found that both channel subtypes form clusters of similar size (Figure 3—figure supplement 1B–F). The mean areas of Ca_V_1.3_L_ and Ca_V_1.3_S_ channel clusters were 2543 ± 50 nm^2^ and 2119 ± 73 nm^2^, respectively (Figure 3—figure supplement 1G, n = 7). GSD images were acquired in the TIRF focal plane with a penetration depth of 130 nm.

To determine the number of channels within Ca_V_1.3 channel clusters, we used step-photobleaching (Ulbrich and Isacoff, 2007), of expressed green fluorescent protein (GFP)-fused Ca_V_1.3_S_ channels in hippocampal neurons (Figure 3E and F) and tsA-201 cells (Figure 3—figure supplement 1G and H). The rationale for using only Ca_V_1.3_S_-GFP channels in these studies is twofold. *First*, Ca_V_1.3_S_ and not Ca_V_1.3_L_ channels have the ability to undergo coupled activation. *Second*, the size of Ca_V_1.3_L_ and Ca_V_1.3_S_ puncta (at least in tsA-201 cells) is similar. Thus, Ca_V_1.3_L_ and Ca_V_1.3_S_ clusters are likely composed of the same number of channels. We identified and excited single Ca_V_1.3_S_ channel clusters in hippocampal neurons and tsA-201 cells using TIRF microscopy with a penetration depth of 130 nm. Although some intracellular signal could be collected, the use of TIRF restricts the signal mainly to the plasmalemmal fraction of the channels. After continuous photobleaching we counted the number of step-wise decreases in fluorescence intensity of Ca_V_1.3_S_-GFP clusters. The majority (65%) of Ca_V_1.3_S_ clusters underwent at least five stepwise decreases in fluorescence, with the remaining clusters showing fewer steps. A single photobleaching step was observed in only 4% of the Ca_V_1.3_S_ clusters. The mean number of Ca_V_1.3_S_ channels per cluster determined through sequential photobleaching was 8 ± 1 (n = 1105 clusters, 18 cells) in hippocampal neurons, this number is consistent with the average cluster area calculated from our super-resolution data and is similar to that reported in two different studies on the L-type channel distribution in hippocampal neurons using immunogold-labeling with electron microscopy and high-resolution immunofluorescence techniques (Leitch et al., 2009; Obermair et al., 2004). Step-photobleaching in tsA-201 cells revealed an average of 5 ± 1 Ca_V_1.3_S_ channels per cluster (n = 585 clusters, 11 cells), consistent also with our super-resolution cluster area measurements (Figure 3—figure supplement 2).

Collectively, these results support our working hypothesis that multi-quantal events detected by imaging Ca^2+^ influx represent the simultaneous activity of multiple Ca_V_1.3_S_ channels in a membrane microdomain. Furthermore, our data suggest that although channel clustering may be necessary for functional coupling of adjacent Ca_V_1.3_S_ channels, physical clustering alone is not sufficient to induce functional coupling in Ca_V_1.3_L_ channels.

### Physical interaction between Ca_V_1.3_S_ channels induces *I_Ca_* facilitation

**Figure 4.**
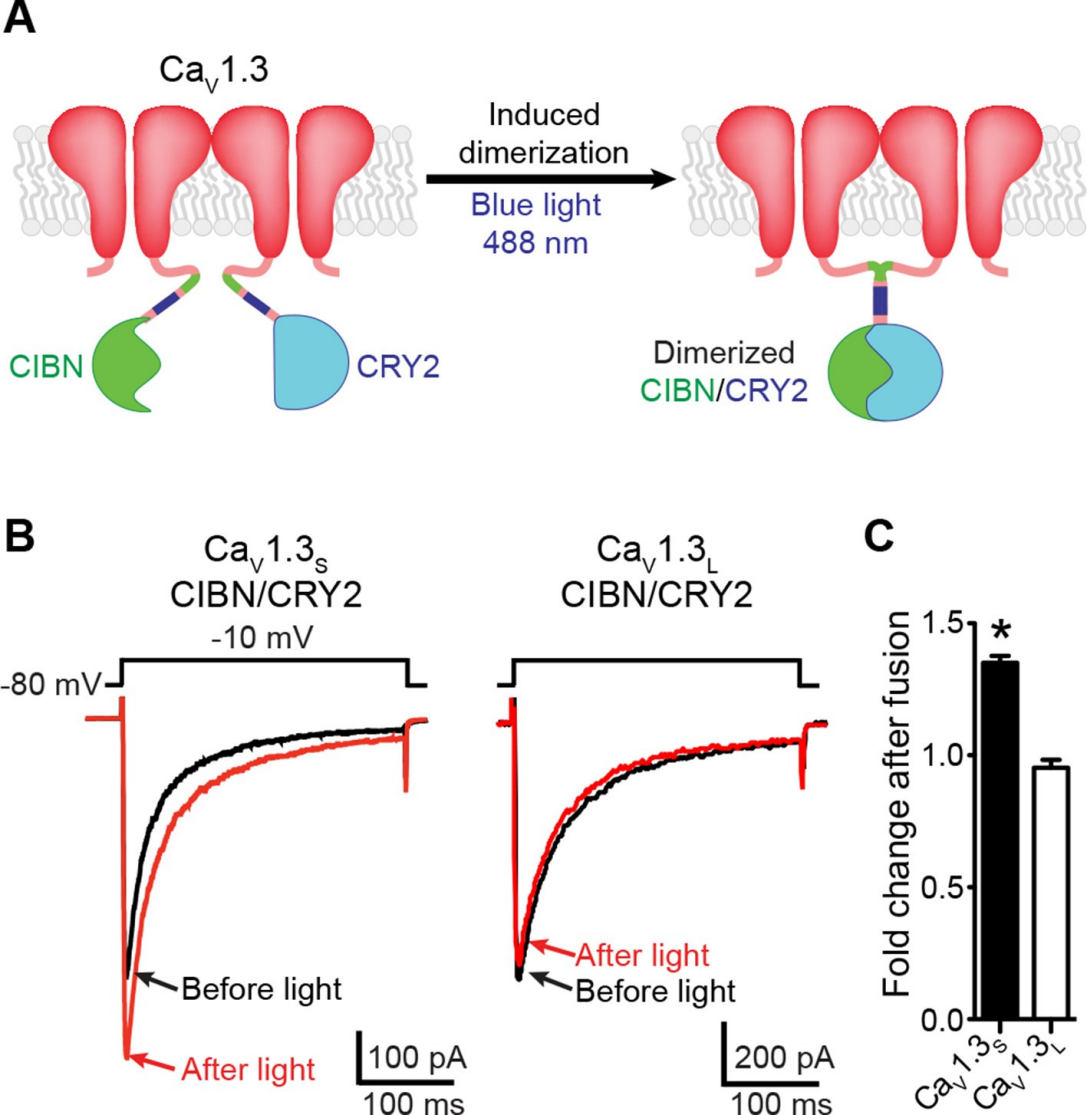
Light-induced fusion increases I_Ca_ amplitude in Ca_V_1.3_S_ but not in Ca_V_1.3_L_ channels. (**A**) Schematic of the blue light-induced dimerization system (CIBN-CRY2) fused to the C-terminal of Ca_V_1.3_S_ channels. The same proteins were fused to the C-terminal of Ca_V_1.3_L_ channels (not shown in schematic). (**B**) Representative current records from tsA-201 cells expressing Ca_V_1.3_S_-CIBN/Ca_V_1.3_S_-CRY2 (*left*) or Ca_V_1.3_L_-CIBN/Ca_V_1.3_L_-CRY2 (*right*), before (*black traces*) and after (*red traces*) induction of channel coupling by excitation with a 30 s pulse of 488 nm light. (**C**) Bar plot of the averaged fold-change in *I_Ca_* following 488 nm excitation in cells expressing Ca_V_1.3_S_-CIBN/Ca_V_1.3_S_-CRY2 (black) or Ca_V_1.3_L_-CIBN/Ca_V_1.3_L_-CRY2. Bars are averages of 5 cells ± SEM (*p<0.05). **DOI:** http://dx.doi.org/10.7554/eLife.15744.009

To determine whether a physical interaction between Ca_V_1.3_S_ channels induces *I_Ca_*facilitation, we fused the C-terminus of Ca_V_1.3_S_ and Ca_V_1.3_L_ channels to an optogenetic light-induced dimerization system based on CIBN and CRY2 proteins (Kennedy et al., 2010). Blue-light illumination (488 nm), promotes CIBN and CRY2 fusion, which forces the C-termini of the attached channels to interact (Figure 4A).

We transfected tsA-201 cells with Ca_V_1.3_S_-CIBN and Ca_V_1.3_S_-CRY2 or Ca_V_1.3_L_-CIBN and Ca_V_1.3_L_-CRY2 channels and measured *I_Ca_* in response to a series of depolarizing voltage steps before and after a 30-s exposure to blue light (488 nm) (Figure 4B). As shown in Figure 4C, in cells expressing Ca_V_1.3_S_-CIBN and Ca_V_1.3_S_-CRY2 channels, *I_Ca_* amplitude increased by 35% (n = 6, p<0.001) after illumination, whereas in cells expressing Ca_V_1.3_L_-CIBN and Ca_V_1.3_L_-CRY2 channels, there was no change in current amplitude (0.95 ± 0.03 n = 6). These results suggest that fusing adjacent channels at the tip of their C-tail increase the probability of functional coupling between Ca_V_1.3_S_ adjoined channels but not between Ca_V_1.3_L_ channels.

### Ca_V_1.3_S_ channels couple under membrane depolarization in a Ca^2+^-dependent manner

**Figure 5.**
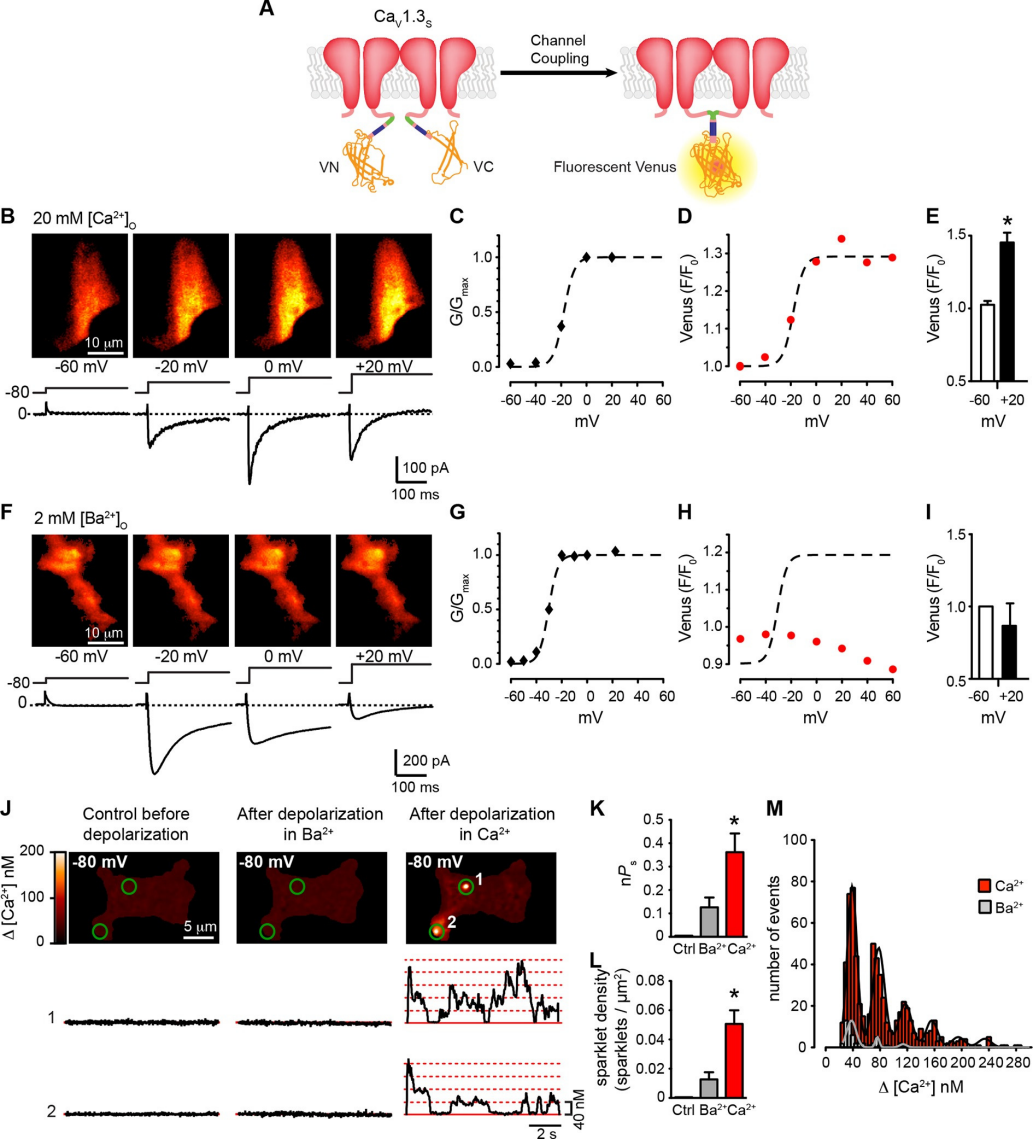
Coupling of Ca_V_1.3_S_ channels is Ca^2+^-dependent and increases channel activity. (**A**) Schematic of Ca_V_1.3_S_ fused to VN and VC fragments of the Split Venus bimolecular fluorescence complementation system. (**B**) TIRF images of Venus fluorescence reconstitution in the presence of 20 mM Ca^2+^ in tsA-201 cells expressing Ca_V_1.3_S_-VN and Ca_V_1.3_S_-VC (*top*). Fluorescence reconstitution was measured in response to 9-s depolarizing voltage steps from a holding potential of -80 mV to test potentials of -60 mV to +60 mV. *I_Ca_* currents evoked at the different voltage steps (*bottom*). (**C**) Voltage-dependence of the normalized conductance (G/G_max_) of the *I_Ca_*shown in (**B**). Dashed curve is the fit to a Boltzmann function. (**D**) Voltage-dependence of Venus fluorescence reconstitution in the presence of 20 mM Ca^2+^. The Boltzmann function calculated in (**C**) is superimposed to compare voltage-dependence. (**E**) Bar plot of averaged Venus fluorescence in the presence of 20 mM Ca^2+^ at -60 mV and +20 mV. Bars are averages ± SEM (*p<0.05, n = 5 cells). (**F**) TIRF images of Venus fluorescence reconstitution in the presence of 2 mM Ba^2+^ in tsA-201 cells expressing Ca_V_1.3_S_-VN and Ca_V_1.3_S_-VC (*top*). Format and protocol are as in (**B**) *I_Ba_ *currents evoked at the different voltage steps (*bottom*). (**G**) Voltage dependence of normalized conductance (G/G_max_) of the *I_Ba_*shown in (**F**). Dashed curve is the fit to a Boltzmann function. (**H**) Voltage dependence of Venus fluorescence reconstitution in the presence of 2 mM Ba^2+^. The Boltzmann function calculated in (**G**) was superimposed to compare voltage-dependence. (**I**) Bar plot of averaged Venus fluorescence in the presence of 20 mM Ca^2+^ at -60 mV and +20 mV. Bars are averages of 5 cells ± SEM (*p<0.05). Venus reconstitution was also tested in the presence 2 mM Ca^2+^ (See Figure 5—figure supplement 1) (**J**) Top: TIRF images of Ca_V_1.3_S_ sparklets recorded at -80 mV in 20 mM Ca^2+^, before depolarization (*left*), after the same depolarization protocol used in (**B, F**) in the presence of 2 mM Ba^2+^ (*center*), and after depolarization in the presence of 20 mM Ca^2+^ (*right*). Green circles indicate sparklet sites. Bottom: Traces of the time course of [Ca^2+^]_i_ in sites 1 and 2 under the three conditions. (**K**) Bar plot of the averaged Ca_V_1.3_S_ sparklet activity (*nPs*) before depolarization (black; average is ~0), after depolarization in Ba^2+^ (gray), and after depolarization in Ca^2+^ (red). Bars are averages ± SEM (*p<0.05, n = 5 cells). (**L**) Bar plot of sparklet density. Format as in (**K**). (**M**) Event amplitude histograms of Ca_V_1.3_S_ sparklets recorded after depolarization in the presence of Ba^2+^ (gray) or Ca^2+^ (red). The amplitude of elementary Ca_V_1.3 sparklets was calculated by fitting histograms to a multicomponent Gaussian function. The experiments in this figure were performed using the Ca_V_1.3_S_ channel encoded by the Addgene plasmid 26576, similar results for split Venus reconstitution and sparklet activity were observed for the plasmid 49,333 (Figure 5—figure supplement 2). **DOI:** http://dx.doi.org/10.7554/eLife.15744.010

We used a second optogenetic approach that entailed fusing Ca_V_1.3_S_ and Ca_V_1.3_L_ channels with either the N- (VN) or C-terminus (VC) of the Venus fluorescent protein (Kodama and Hu, 2010; Shyu et al., 2006) (Figure 5A). Individual VN and VC fragments are non-fluorescent, but when they come into close proximity, they can reconstitute a full Venus protein, resulting in fluorescence. Venus reconstitution is irreversible and thus, the intensity of the fluorescence signal increases with time, proportionate to the number of Ca_V_1.3_S/L_-VN and Ca_V_1.3_S/L_-VC channels that physically associate.

**Figure 5—figure supplement 1.**
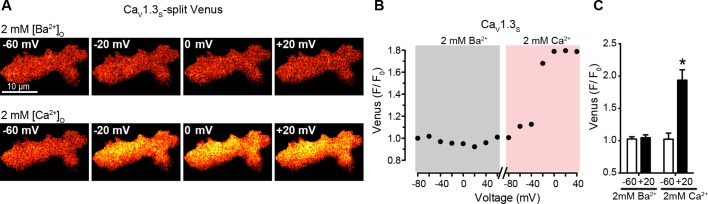
Depolarization in the presence of physiological Ca^2+^ concentrations induces coupling in Ca_V_1.3_S_. (**A**) TIRF images of Venus fluorescence reconstitution in the presence of 2 mM Ba^2+^ in tsA-201 cells expressing Ca_V_1.3_S_-VN and Ca_V_1.3_S_-VC (*top*). TIRF images of Venus fluorescence reconstitution in the presence of 2 mM Ca^2+^ in the same cell (*bottom*). Fluorescence reconstitution was measured as described in Figure 5. (**B**) Voltage-dependence of Ca_V_1.3_S_-VN and Ca_V_1.3_S_-VC Venus fluorescence reconstitution in the presence of 2 mM Ba^2+^ followed by 2 mM Ca^2+^. (**C**) Bar plot of averaged Venus fluorescence in the presence of 2 mM Ba^2+^ and 2 mM Ca^2+^ at -60 mV and +20 mV. Bars are averages ± SEM (*p<0.05, n = 6 cells). Bars are averages ± SEM (*p<0.05, n = 6 cells). **DOI:** http://dx.doi.org/10.7554/eLife.15744.011

**Figure 5—figure supplement 2.**
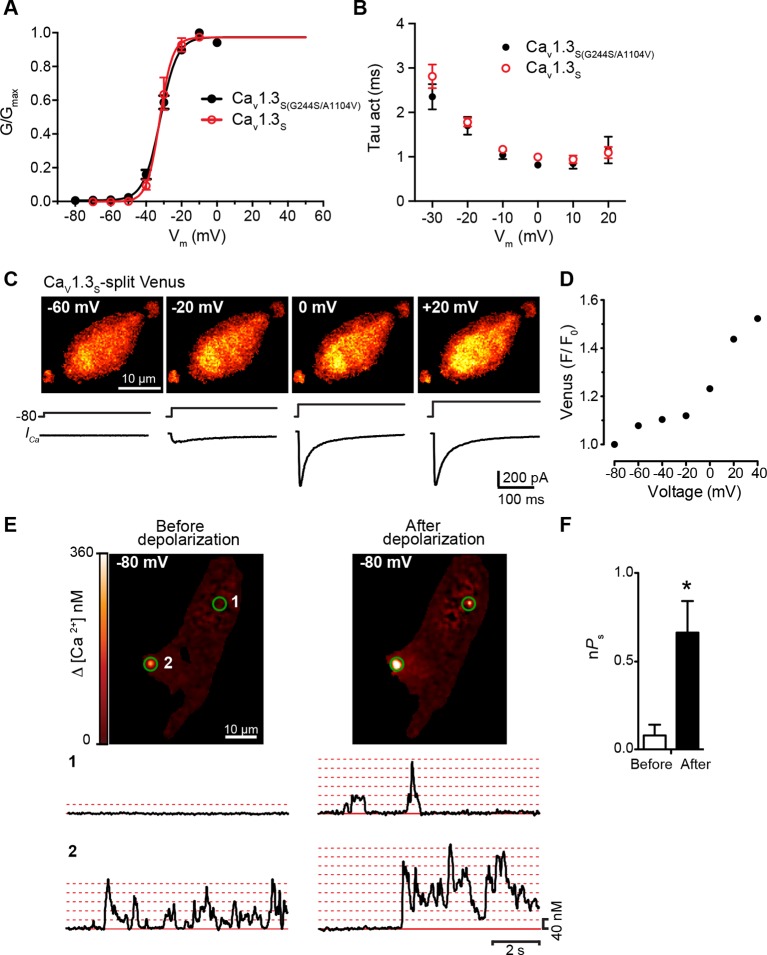
Ca_V_1.3_S_ and Ca_V_1.3_S(G244S)_ channels exhibit Ca^2+^-dependent coupling and increased sparklet activity after depolarization. (**A**) Voltage-dependence of the normalized conductance (G/G_max_) of the *I_Ca_ *from tsA-201 cells expressing Ca_V_1.3_S_ (Addgene 49333) or Ca_V_1.3_S(G244S/A1104V)_ (Addgene 26576). (**B**) Plot of the time constants of activation against the depolarization voltage. Current traces were fitted by a single exponential and the corresponding time constants were averaged and plotted in the graph. Data from 6 cells per group. (**C**) TIRF images of Venus fluorescence reconstitution in the presence of 2 mM Ca^2+^ in tsA-201 cells expressing Ca_V_1.3_S_ (*top*). Fluorescence reconstitution was measured in response to 9-s depolarizing voltage steps from a holding potential of -80 mV to test potentials of -60 mV to +60 mV. *I_Ca_* currents evoked at the different voltage steps (*bottom*). (**D**) Voltage-dependence of Venus fluorescence reconstitution in the presence of 2 mM Ca^2+^. (**E**) (*top*) TIRF images of Ca_V_1.3_S_ sparklets recorded at -80 mV in 20 mM Ca^2+^, before depolarization (*left*), after the same depolarization protocol used in (**C**) in the presence of 20 mM Ca^2+^ (*right*). Green circles indicate sparklet sites. (*Bottom*)Traces of the time course of [Ca^2+^]_i_ in sites 1 and 2. (**F**) Bar plot of the averaged Ca_V_1.3_S_ sparklet activity (*nPs*) before and after. Bars are averages of 4 cells ± SEM (*p<0.05). **DOI:** http://dx.doi.org/10.7554/eLife.15744.012

We simultaneously recorded *I_Ca_* and obtained TIRF microscopic images from tsA-201 cells transfected with Ca_V_1.3_S_ -VN and Ca_V_1.3_S_ -VC. The first set of experiments was performed in the presence of 20 mM Ca^2+^ to mimic the experimental conditions used to record Ca^2+^ sparklets below. With 20 mM Ca^2+^ in the bathing solution, *I_Ca_* and Venus fluorescence for Ca_V_1.3_S_ channels increased in parallel in response to depolarizing pulses from a holding potential of -80 mV (Figure 5B). The normalized conductance and Venus fluorescence exhibited similar sigmoidal voltage dependencies (Figures 5C–D), which can be attributed to the irreversible nature of the Venus reconstitution, resulting in an increased number of fluorescent proteins with each successive depolarization as the open probability of Ca_V_1.3_S_ channels increases.

Substituting 2 mM Ba^2+^ for 20 mM Ca^2+^ in the external solution led to a robust *I_Ba_* during membrane depolarization, but caused no accompanying change in Venus fluorescence (Figures 5F–I). Importantly, Venus reconstitution was never observed in cells expressing Ca_V_1.3_L_-VN and Ca_V_1.3_L_-VC in (see Figure 8). These data indicate that Ca_V_1.3_S_ channels, but not Ca_V_1.3_L_ channels, can physically interact via their C-termini, that this association occurs in response to membrane depolarization, and that it is promoted by intracellular Ca^2+^.

Because Venus reconstitution is irreversible, once Ca_V_1.3_S_-VN and Ca_V_1.3_S_-VC channels fuse they must remain adjoined. Thus, we tested the hypothesis that Ca^2+^-induced fusion of Ca_V_1.3_S_-VN and Ca_V_1.3_S_-VC increases the activity of adjoined channels by recording Ca_V_1.3_S_ sparklets at a holding potential of -80 mV, in the presence of Ba^2+^ or Ca^2+^, before and after applying the same depolarization protocol described above. Before depolarization, Ca_V_1.3_S_ sparklet activity was very low (Figure 5J); after depolarization, Ca_V_1.3_S_ sparklet activity (n*P*_s_; Figure 5K) and sparklet density (Figure 5L) markedly increased in the presence of Ca^2+^, but not Ba^2+^, without a change in the amplitude of the elementary Ca^2+^ influx (Figure 5M).

### Functional Ca_V_1.3_S_-to-Ca_V_1.3_S_ channel coupling is mediated by physical interactions between Ca^2+^-CaM and the pre-IQ domain

**Figure 6.**
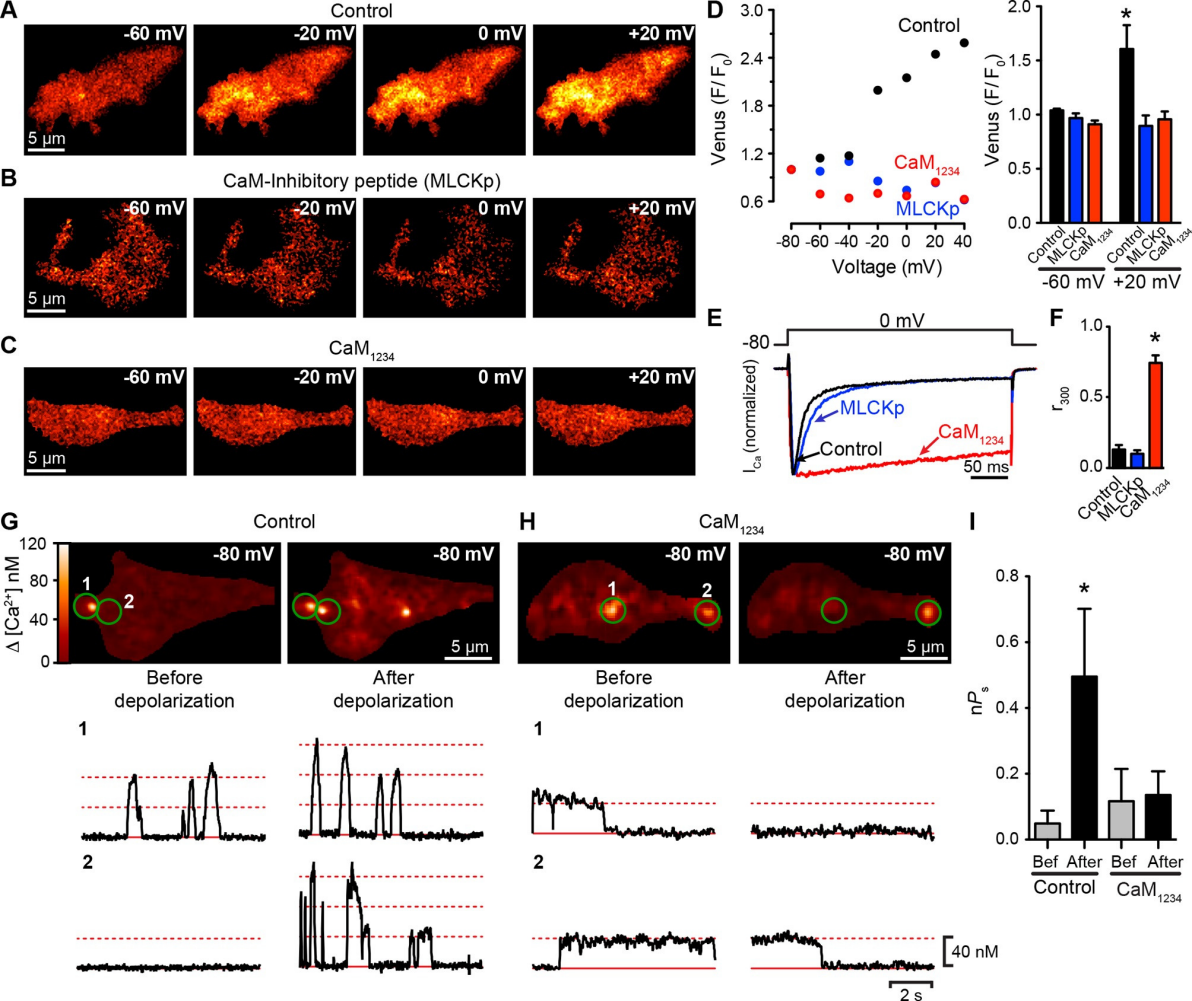
Ca_V_1.3_S_ coupling requires Ca^2+^-CaM. (**A–C**) TIRF images of Venus fluorescence reconstitution in the presence of 20 mM Ca^2+^ in tsA-201 cells expressing (**A**) Ca_V_1.3_S_-VN and Ca_V_1.3_S_-VC, (**B**) Ca_V_1.3_S_-VN and Ca_V_1.3_S_-VC and dialyzed with the MLCK peptide (MLCKp), (**C**) Ca_V_1.3_S_-VN, Ca_V_1.3_S_-VC and CaM_1234_. Fluorescence reconstitution was measured in response to depolarizing voltage steps from a holding potential of -80 mV to test potentials of -60 mV to +60 mV. (**D**) Voltage-dependence of Venus fluorescence reconstitution in the presence of 20 mM Ca^2+^ for control (black), MLCKp (blue), and CaM_1234_ (red) cells shown in (**A–C**) (*left*). Bar plot of averaged Venus fluorescence in the presence of 20 mM Ca^2+^ at -60 mV and +20 mV (*right*). Bars are averages ± SEM (*p<0.05, n = 5 cells). (**E**) Normalized *I_Ca_* currents evoked by a 300-ms depolarizing pulse from a holding potential of -80 mV to a test potential of 0 mV in control (black), MLCKp (blue), and CaM_1234_ (red) cells. Currents analyzed for these experiments were in a range between 0.3 and 1.2 nA (**F**) Bar plot of the mean fraction of r_300_ at 0 mV. Bars are averages ± SEM (*p<0.05, n = 5 cells). (**G**) Top: TIRF images of Ca_V_1.3_S_ sparklets in tsA-201 cells expressing Ca_V_1.3_S_-VN and Ca_V_1.3_S_-VC (Control). Sparklets were recorded at -80 mV in 20 mM Ca^2+^ before depolarization (left) and after the same depolarization protocol used in (**A–C**) (right). Green circles indicate sparklet sites. Bottom: Traces of the time course of [Ca^2+^]_i_ in the corresponding sparklet sites 1 and 2. (**H**) TIRF images and time course of [Ca^2+^]_i_ of Ca_V_1.3_S_ sparklets in tsA-201 cells expressing Ca_V_1.3_S_-VN/Ca_V_1.3_S_-VC and CaM_1234_. Format and protocol are as in (**G**). (**I**) Bar plot of the averaged Ca_V_1.3_S_ sparklet activity (n*Ps*) before (gray) and after (black) depolarization. Bars are averages 5 cells ± SEM (*p<0.05). **DOI:** http://dx.doi.org/10.7554/eLife.15744.013

To investigate the mechanism underlying the Ca^2+^-dependency of Ca_V_1.3_S_ channel coupling further, we used the Venus system in conjunction with a mutagenesis approach, focusing on CaM, which is required for CDF and has been shown to bind Ca^2+^ and associate with the C-terminus of L-type Ca^2+^ channels (Zuhlke et al., 1999). tsA-201 cells were transfected with Ca_V_1.3_S_-VN/Ca_V_1.3_S_-VC and divided into three groups. Cells in the first group (controls) were dialyzed with a standard Cs^+^-based intracellular solution. Cells in the second group were dialyzed with a CaM-inhibitory peptide corresponding to a 15-aa fragment of the wild-type CaM-binding domain of myosin light chain kinase (MLCKp; 1 μM), which binds to CaM with high affinity (apparent dissociation constant, ~6 pM) in the presence of Ca^2+^ (Torok and Trentham, 1994) and has been used by others as a competitive inhibitor of CaM (Ciampa et al., 2011; Mercado et al., 2010; Piper and Large, 2004). The third group consisted of cells co-expressing a dominant-negative mutant form of CaM (CaM_1234_) that does not bind Ca^2+^ through its N- or C-terminal lobes. Dialysis of MLCKp or co-expression of CaM_1234_ prevented Ca_V_1.3_S_-VN and Ca_V_1.3_S_-VC fusion upon membrane depolarization (Figure 6A–D). Although MLCKp and CaM_1234_ were equally effective in preventing Venus reconstitution, they had differential effects on I_Ca_ inactivation (Figure 6E). Whereas the fraction of peak *I_Ca_ *remaining at 300 ms (r_300_) in MLCKp-dialyzed cells (0.11 ± 0.03, n = 5) was similar to that of controls (0.14 ± 0.03, n = 5), the rate of inactivation of *I_Ca_* was slower in cells expressing CaM_1234_, as reflected in a much higher r_300_ value (0.75 ± 0.05, n = 5) (Figure 6F). Our interpretation of these findings is that the CaM molecules involved in CDI are distinct from those involved in functional coupling of Ca_V_1.3_S_ channels. The results suggest that CaM molecules that mediate coupling could be both attached to the channels or recruited to the C-terminus during depolarization. This could explain why they are accessible to MLCKp blockade, unlike the CaM molecules that mediate CDI, which are tethered to the IQ domain of the channels (Pitt et al., 2001).

To extend this analysis, we recorded Ca_V_1.3_S_ sparklets in control and CaM_1234_ cells before and after depolarization to +20 mV (Figure 6G and H). Although multi-quantal Ca_V_1.3_S_ sparklets were observed in control cells at rest (e.g., Figure 6G, trace 2 left), sparklets in CaM_1234_ cells prior to depolarization were long, single-quantal events (Figure 6H, traces 1 and 2, left). These long Ca_V_1.3_S_ sparklets were likely due to decreased CDI of Ca_V_1.3_S_ channels in cells expressing CaM_1234_ (Yang et al., 2014). Importantly, the overall Ca_V_1.3_S_ sparklet density was lower in cells expressing CaM_1234_ than WT, suggesting that Apo-CaM does not increase Ca_V_1.3_S_ channel activity.

The coupling coefficient for Ca_V_1.3_S_ sparklet sites in WT cells was 0.07 ± 06, whereas that in CaM_1234_ cells was 0.02 ± 0.02 (n = 5). Membrane depolarization increased the coupling coefficient of Ca_V_1.3_S_ channels within multi-quantal sparklet sites in control cells (0.18 ± 0.03, n = 5), but not in CaM_1234_ cells (0.08 ± 0.05, n = 5). The opposing effects of CaM_1234_ on Ca_V_1.3_S_ sparklets — longer, but decoupled — resulted in Ca_V_1.3_S_ sparklet activity before depolarization that was similar in control (*nP*_S_ = 0.07 ± 0.05; p>0.05) and CaM_1234_ (*nP*_S_ = 0.12 ± 0.09) cells (Figure 6I). However, depolarization increased Ca_V_1.3 sparklet activity nearly 7-fold in control cells, but had no effect on sparklet activity in CaM_1234_ cells. Taken together with the *I_Ca_* and Venus reconstitution results described above, these data strongly suggest that Ca^2+^ binding to CaM is required for physical and functional Ca_V_1.3_S_-to-Ca_V_1.3_S_ channel coupling.

**Figure 7.**
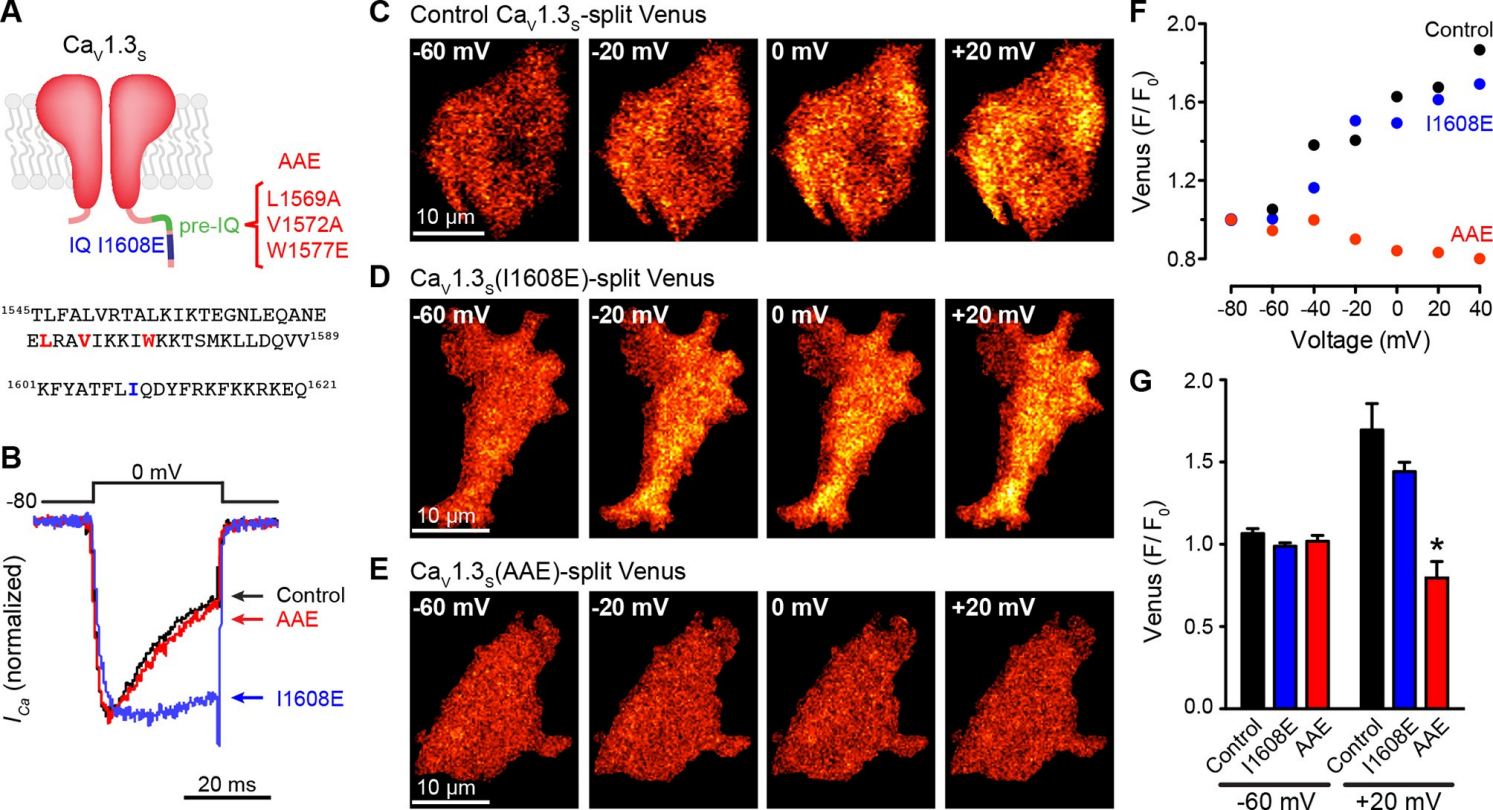
The pre-IQ domain is required for Ca^2+^-CaM-mediated Ca_V_1.3_S_ coupling. (**A**) Schematic of Ca_V_1.3_S_ mutations introduced to disrupt CaM binding to the IQ (I1608E) or the pre-IQ (AAE) domain; the position of the mutated amino acid is shown in the sequence below. (**B**) Normalized *I_Ca_* currents evoked by a 30-ms depolarizing pulse from a holding potential of -80 mV to a test potential of 0 mV in tsA-201 cells expressing Ca_V_1.3_S_ (Control, black), Ca_V_1.3_S_(I1608E) (blue), or Ca_V_1.3_S_(AAE) (red). Currents analyzed for these experiments were in a range between 100 and 600 pA (**C–E**) TIRF images of Venus fluorescence reconstitution in the presence of 20 mM Ca^2+^ in tsA-201 cells expressing (**C**) Ca_V_1.3_S_-VN and Ca_V_1.3_S_-VC, (**D**) Ca_V_1.3_S_(I1608E)-VN and Ca_V_1.3_S_(I1608E)-VC, or (**E**) Ca_V_1.3_S_(AAE)-VN and Ca_V_1.3_S_(AAE)-VC. Fluorescence reconstitution was measured in response to depolarizing voltage steps from a holding potential of -80 mV to test potentials of -60 mV to +60 mV. (**F**) Voltage-dependence of Venus fluorescence reconstitution in the presence of 20 mM Ca^2+^ for control (black), I1608E mutant (blue), and AAE mutant (red) from the cells shown in (**C–E**). (**G**) Bar plot of averaged Venus fluorescence in the presence of 20 mM Ca^2+^ at -60 mV and +20 mV. Bars are averages of 5 cells ± SEM (*p<0.05). **DOI:** http://dx.doi.org/10.7554/eLife.15744.014

Finally, to establish the molecular mechanism by which CaM might mediate these effects, we mutated different CaM-binding domains of the Ca_V_1.3_S_. L-type channels have two binding sites for CaM in their C-terminus: the IQ domain (aa K1601-Q1621) and the pre-IQ domain (aa T1545-Q1587) (Fallon et al., 2009). These sites have different affinities for CaM; whereas CaM is “pre-associated” and binds tightly to the IQ domain, the association of CaM with the pre-IQ domain is seemingly weaker and likely transient. To determine which of these sites is necessary for CaM-mediated Ca_V_1.3_S_ coupling, we generated two mutants of the Ca_V_1.3_S_-VN and Ca_V_1.3_S_-VC channels. The first contained a single point mutation I1608E (Ca_V_1.3-I1608E) that disrupts CaM binding to the IQ domain (Zuhlke et al., 1999), and the second contained a triple mutation (L1569A, V1572A, and W1577E; Ca_V_1.3_S_-AAE) that prevents CaM binding to the pre-IQ domain and anti-parallel coiled-coil arrangement of the pre-IQ domains (Fallon et al., 2009) (Figure 7A). Ca_V_1.3_S_-I1608E channels showed a slower rate of inactivation than control and Ca_V_1.3_S_-AAE channels (Figure 7B), consistent with the lack of CDI. Interestingly, Ca_V_1.3_S_-I1608E-VN/VC, but not Ca_V_1.3_S_-AAE VN/VC, which were capable of reconstituting Venus during membrane depolarization (Figure 7C–G). These data suggest that binding of CaM to the pre-IQ domain is critically involved in Ca_V_1.3_S_ channel coupling during membrane depolarization and further supports our previous assertion that the CaM pool involved in CDI is distinct from that involved in channel coupling.

### Deletion of DCRD is not sufficient to allow coupling in Ca_V_1.3_L_ channels

**Figure 8.**
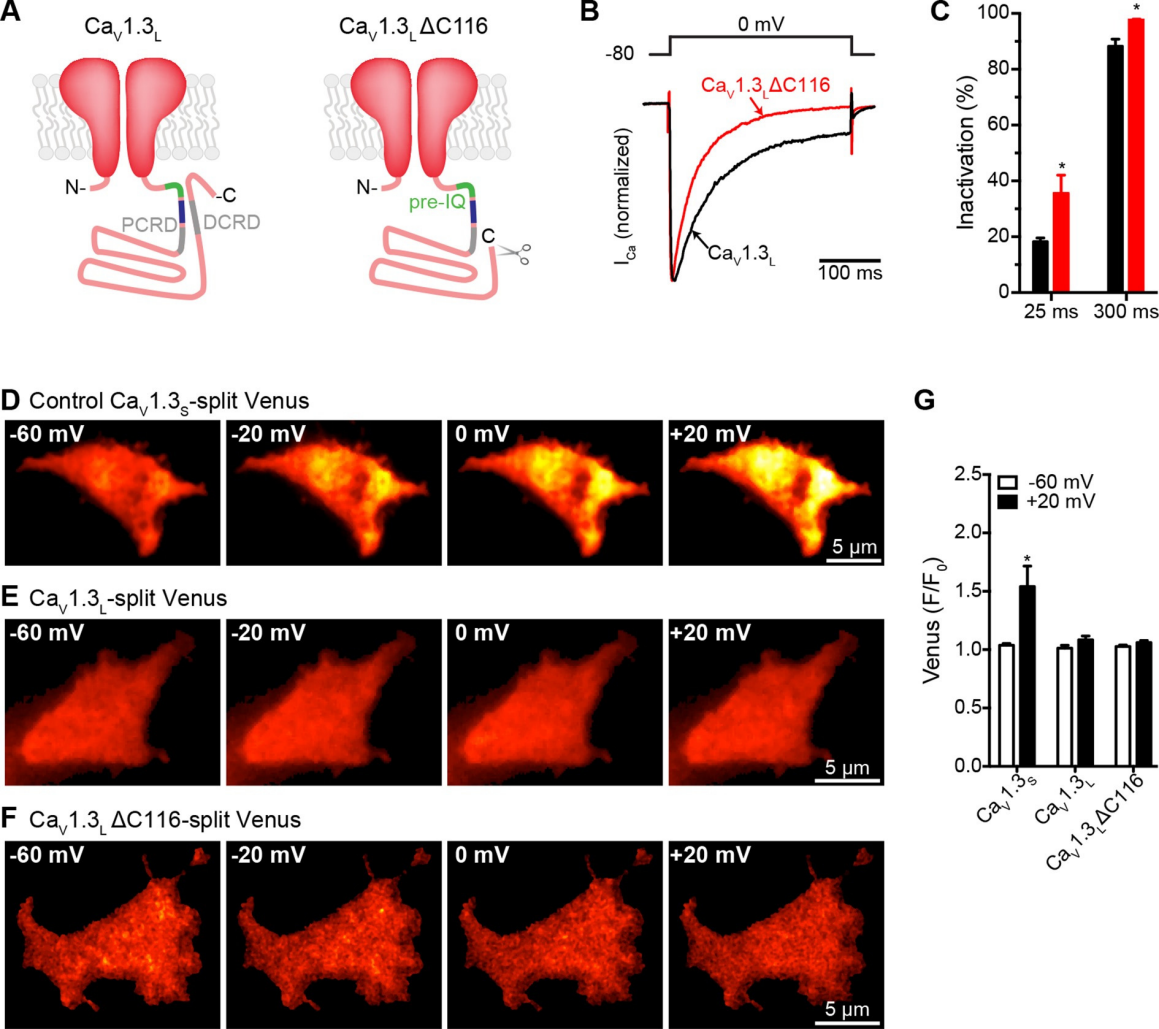
Distal auto-regulatory domain (DCRD) is not responsible for the lack of coupling of Ca_V_1.3_L_ channels. (**A**) Schematic of Ca_V_1.3_L_ channel splice variant (*left*), depicting the domains important for Ca^2+^-mediated regulation: pre-IQ (green), IQ (blue), proximal and distal C-terminal regulatory domains (PCRD, DCRD, gray. Schematic of the Ca_V_1.3_L_ΔC116 channel where the last 116 aa in the C-terminal were removed (*right*). (**B**) Representative currents of Ca_V_1.3_L_ (black) and Ca_V_1.3_L_ ΔC116 channels (red) expressed in tsA-201 cells. Currents were evoked by a 300-ms depolarization from holding potential of -80 mV to a test potential of 0 mV, with 2 mM Ca^2+^ as the charge carrier. Currents analyzed for these experiments were in a range between 0.3 and 1 nA (**C**) Bar plot of the% inactivation after 25 or 300 ms at 0 mV. Bars are averages of 5 cells ± SEM (*p < 0.001) (**D–F**) TIRF images of Venus fluorescence reconstitution in the presence of 2 mM Ca^2+^ in tsA-201 cells expressing Ca_V_1.3_S_-VN and Ca_V_1.3_S_-VC (**D**) Ca_V_1.3_L_-VN and Ca_V_1.3_L_-VC (**E**) or Ca_V_1.3_L_ ΔC116-VN and Ca_V_1.3_L_ ΔC116-VC (**E**). Fluorescence reconstitution was measured in response to depolarizing voltage steps from a holding potential of -80 mV to test potentials of -60 mV to +60 mV. (**G**) Bar plot of averaged Venus fluorescence at -60 mV and +20 mV for each of the aforementioned construct pairs. Bars are averages of 5 cells ± SEM (*p<0.05). Data for Ca_V_1.3_L_ ΔC116-VN and Ca_V_1.3_L_ ΔC116-VC Venus reconstitution with 20 mM Ca^2+^ is presented in Figure 8—figure supplement 1. **DOI:** http://dx.doi.org/10.7554/eLife.15744.015

We investigated one of the possible molecular mechanisms that prevent functional coupling in Ca_V_1.3_L_ channels. Singh et al (2008) showed that a Ca_V_1.3_L_ mutant lacking the last 116 amino acids of the C-terminus (Ca_V_ 1.3_L_∆116) had voltage-dependencies and kinetics similar to those of Ca_V_1.3_S_ channels. The Ca_V_1.3_L_∆116 channels lack the distal regulatory domain (DCRD) that binds the proximal regulatory domain (PCRD) located downstream to the pre-IQ and IQ domains of the channel (Figure 8A). Interaction of these two regulatory domains interferes with CaM binding to the IQ domain and results in a reduction in CDI (Bock et al., 2011; Singh et al., 2008). If the DCRD interferes also with the binding of CaM to the pre-IQ domain, which we propose is important for channel-to-channel coupling, we would expect that removing the DCRD would allow coupling between Ca_V_1.3_L_ channels. Thus, we investigated whether or not Ca_V_1.3_L_∆116 channels are capable of undergoing Ca^2+^-driven physical interactions. For these experiments, we created Ca_V_1.3_L_∆116 channels fused to the split Venus system. As expected, CDI of *I_Ca_* was faster in cells expressing the Ca_V_1.3_L_∆116 channels compared to the full length Ca_V_1.3_L_ channels (p<0.05; Figure 8B and C).

**Figure 8—figure supplement 1.**
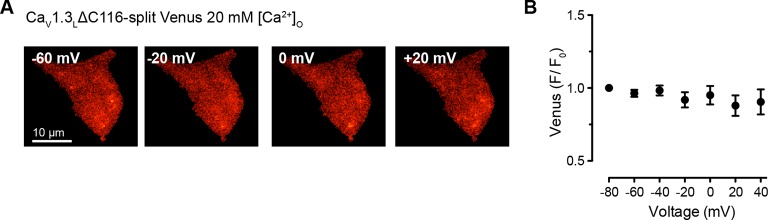
High Ca2+ concentration is not enough to induce coupling in Ca_V_1.3_L_ΔC116 channels. (**A**) TIRF images of Venus fluorescence reconstitution in the presence of 20 mM Ca^2+^ in tsA-201 cells expressing Ca_V_1.3_L_ ΔC116-VN and Ca_V_1.3_L_ ΔC116-VC. Fluorescence reconstitution was measured in response to depolarizing voltage steps from a holding potential of -80 mV to test potentials of -60 mV to +60 mV. (**B**) Voltage-dependence of Venus fluorescence reconstitution in the presence of 20 mM Ca^2+^. Points are averages of 5 cells ± SEM. **DOI:** http://dx.doi.org/10.7554/eLife.15744.016

We found that, with 2 mM Ca^2+^ in the bathing solution, cells expressing Ca_V_1.3_L_∆116 channels failed to reconstitute Venus fluorescence, similar to what we observed for the full length Ca_V_1.3_L_ channel (Figure 8D–G). Even increasing the extracellular Ca^2+^ concentration to 20 mM was not enough to induce coupling in the Ca_V_1.3_L_∆116 channels (Figure 8—figure supplement 1, *n* = 5, p=0.245 -60 mV vs 20 mV). This result suggests that deletion of DCRD is not sufficient to allow coupling of Ca_V_1.3_L_ channels. As Ca_V_1.3_L_∆116 channels still have a C-terminus (396 aa) that is considerably longer than that of Ca_V_1.3_S_ channels, it is possible that another domain inside this region might be responsible for occluding the binding of CaM to the pre-IQ domain, and preventing Ca_V_1.3_L_∆116 channel coupling. Differential folding between the short and long C-terminal could be another explanation for the ability of Ca_V_1.3_S_ channel-to-channel coupling during depolarization-induced Ca^2+^ entry.

### Coupling of Ca_V_1.3_S_ channels increases the firing rate of hippocampal neurons

**Figure 9.**
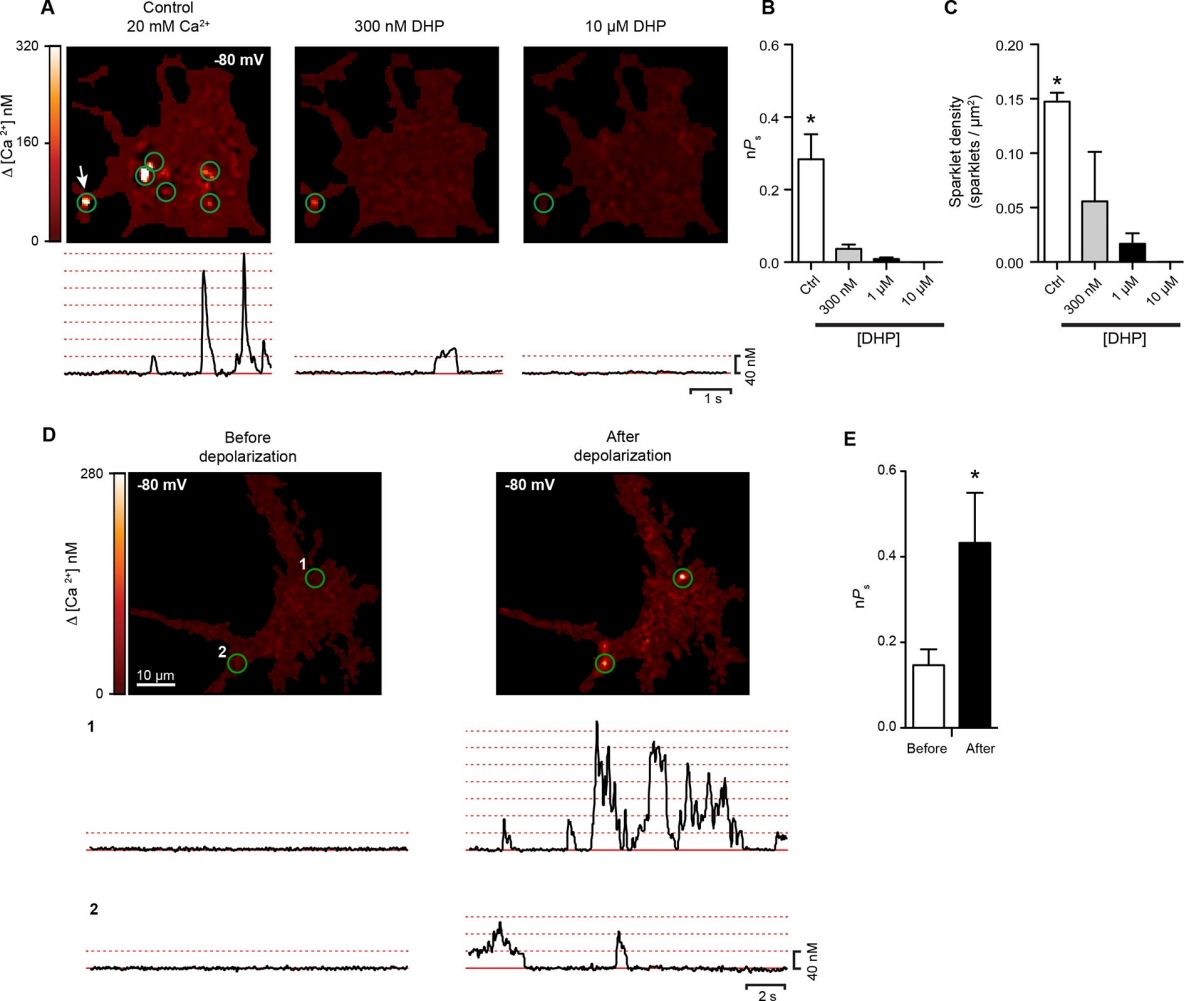
Hippocampal neurons exhibit dihydropyridine-sensitive spontaneous persistent sparklet activity that is increased after depolarization. (**A**) *Top:* TIRF images of Ca^2+^sparklets recorded at -80 mV in cultured hippocampal neurons (4 div) under control conditions (20 mM [Ca^2+^]_o_; *left*), and after exposure to low (1 µM; *middle*) and high (10 µM; *right*) concentrations of dihydropyridine (DHP). Green circles indicate sparklet sites. Bottom: Traces of the time course of [Ca^2+^]_i_ at the site indicated by the white arrow for each condition are shown below the relevant image. Dotted red lines show the amplitudes of 1 to 7 quantal levels. (**B**) Bar chart showing the mean Ca^2+^ sparklet activity (*nPs*) in control conditions and after exposure to 300 nM, 1 µM or 10 µM concentrations of DHP. (**C**) Bar chart showing sparklet density for each condition described in (**B**) (*p<0.05, n = 6 cells). (**D**) *Top*: Ca^2+^ sparklets recorded at -80 mV in cultured hippocampal neurons (4 div), before depolarization (*left*) and after depolarization (*right*). Green circles indicate sparklet sites. Bottom: Traces of the time course of [Ca^2+^]_i_ in sites 1 and 2 before and after depolarization. (**E**) Bar plot of the averaged Ca^2+^ sparklet activity (*nPs*) before (white) and after depolarization (black). Bars are averages of 4 cells ± SEM (*p<0.05). **DOI:** http://dx.doi.org/10.7554/eLife.15744.017

We extended our investigation of the functional consequences of Ca_V_1.3_S_ channel coupling to cultured rat hippocampal neurons. We began by recording spontaneous Ca_V_1.3 sparklets from these cells. As in tsA-201 cells expressing Ca_V_1.3_S_ channels, Ca^2+^ sparklets in neurons were restricted to specific sites and had multi-quantal amplitudes resulting from the simultaneous opening and closing of multiple channels. The quantal unit of Ca^2+^ influx was about 40 nM. The coupling coefficient (κ) of sparklet sites ranged from 0.14 to 0.33. The average κ value was 0.23 ± 0.04 (n = 6). Importantly, sparklet site activity was decreased by application of 300 nM of the dihydropyridine antagonist isradipine and completely eliminated when the concentration of the drug was increased to 10 µM (Figure 9A and B). This is consistent with the hypothesis that sparklets in hippocampal neurons were produced by L-type calcium channels. Both Ca_V_1.2 and Ca_V_1.3 channels are expressed in hippocampal neurons (Hell et al., 1993) and although it is impossible to distinguish between these two L-type Ca^2+^ channels using either electrophysiological or pharmacological methods, it has been shown that Ca_V_1.3 channels have a reduced sensitivity to dihydropyridines compared to Ca_V_1.2 channels (Lipscombe et al., 2004; Xu and Lipscombe, 2001). A previous study by Koschak et al found that 100% of Ca_V_1.2 channels but only ~60% of Ca_V_1.3 channels are inhibited by 300 nM isradipine (Koschak et al., 2001), given this, it is reasonable to assume that any sparklet remaining after application of 300 nM isradipine is more likely to be generated by Ca_V_1.3channels than Ca_V_1.2. In addition, near by 25% of the L-type current in hippocampal neurons is carried by Ca_V_1.3 channels (Moosmang et al., 2005), this proportion is in agreement with the remaining sparklet site density we observed after the treatment with 300 nM isradipine (Figure 9C).

As was the case for tsA-201 cells expressing Ca_V_1.3_S_ channels, we found that conditioning membrane depolarization to 0 mV increased sparklet activity nearly 3-fold in the hippocampal neurons and induced persistent sparklet activity (Figure 9D and E). The coupling coefficient increased from 0.15 ± 0.04 to 0.43 ± 0.12 after membrane depolarization (n = 8). These results support the hypothesis that L-type channels undergo cooperative gating, generating persistent Ca^2+^ influx in hippocampal neurons. The persistent L-type channels sparklet activity evoked by membrane depolarization in the presence of Ca^2+^ bears a striking resemblance to the persistent cationic currents observed in several types of neurons (Fransen et al., 2006; Major and Tank, 2004; Moritz et al., 2007; Powers and Binder, 2001).

**Figure 10.**
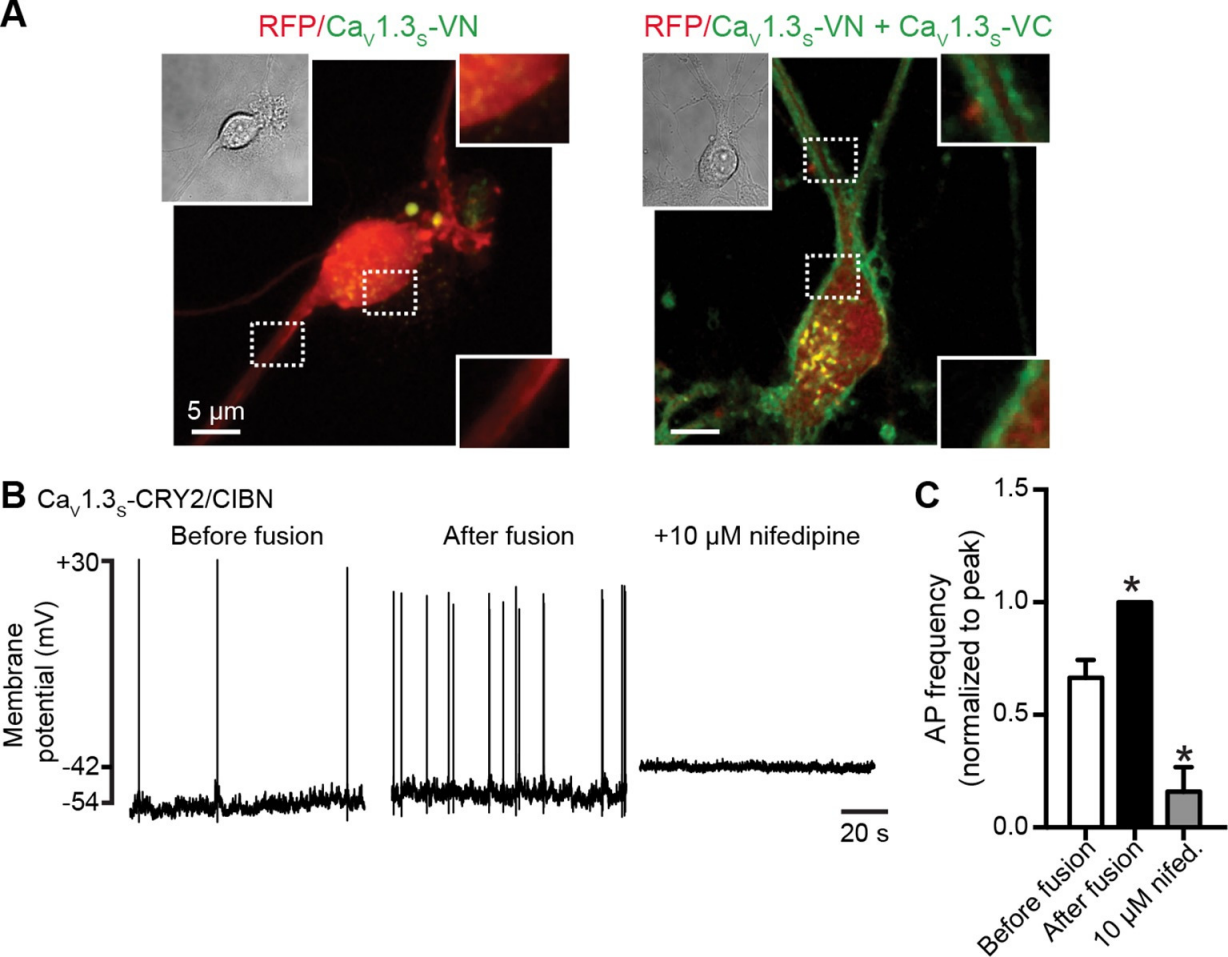
Ca_V_1.3_S_ coupling increases the firing rate of hippocampal neurons. (**A**) Confocal images of two representative cultured hippocampal neurons expressing tRFP as a transfection marker (red) and Ca_V_1.3_S_-VC (*left*, negative control) or Ca_V_1.3_S_-VC/Ca_V_1.3_S_-VN (*right*). Fluorescence of the spontaneously reconstituted Venus is shown in green. The insets show expanded views of the soma and dendritic regions marked by the dashed boxes. Overexpression of these channels does not change the cluster size observed with super-resolution microscopy (see also Figure 10—figure supplemental 1). (**B**) Representative traces of spontaneous action potentials recorded from neurons expressing Ca_V_1.3_S_-CRY2 and Ca_V_1.3_S_-CIBN before (*left*) and after (*middle*) the induction of fusion with 488 nm light and after subsequent treatment with 10 µM nifedipine (*right*). (**C**) Bar plot showing the AP frequency (normalized to the peak frequency) for each condition. Bars are averages of 4 cells ± SEM (*p<0.01). **DOI:** http://dx.doi.org/10.7554/eLife.15744.018

We found that the somatic and dendritic membranes of neurons expressing both Ca_V_1.3_S_-VN and Ca_V_1.3_S_-VC channels displayed prominent Venus fluorescence (Figure 10A), indicating fusion of Ca_V_1.3_S_-VN and Ca_V_1.3_S_-VC channel pairs and functional Venus reconstitution. Since Venus reconstitution is irreversible and neurons have spontaneous electrical activity, this observation suggests that the C-termini of Ca_V_1.3_S_ channels make contact during normal neuronal firing. Although spontaneous self-assembly between Venus subunits might conceivably drive an interaction that would not otherwise occur, the improved Venus system used here has a mutation (I152L) that minimizes non-specific interactions (Kodama and Hu, 2010).

**Figure 10—figure supplement 1.**
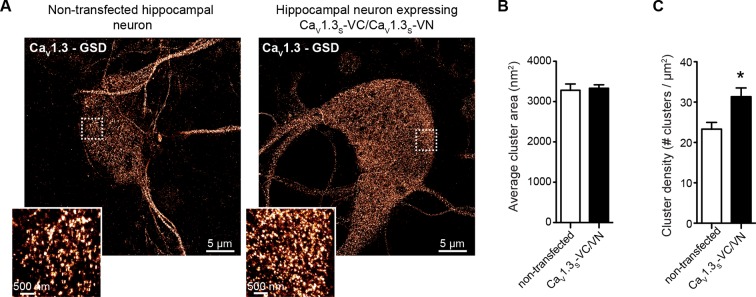
Ca_V_1.3_S_ overexpression in hippocampal neurons increased the number of Ca_V_1.3_S_ channels, but not the cluster size. (**A**) Super-resolution (GSD) image of Ca_V_1.3 channels in representative hippocampal neurons non-transfected (left) or expressing Ca_V_1.3_S_-VN and Ca_V_1.3_S_-VC (right), immunostained against Ca_V_1.3. Insets at the left corner are magnifications of the outlined regions. (**B**) Average cluster area for Ca_V_1.3 channels in non-transfected (white) and Ca_V_1.3_S_-VN and Ca_V_1.3_S_-VC transfected neurons. Bars are averages ± SEM (n = 5 cells). (**C**) Ca_V_1.3 channels cluster density in non-transfected (white) and Ca_V_1.3_S_-VN and Ca_V_1.3_S_-VC transfected neurons. Bars are averages of 5 cells ± SEM (*p<0.05). **DOI:** http://dx.doi.org/10.7554/eLife.15744.019

We analyzed super resolution images of hippocampal neurons transfected with Ca_V_1.3_S_-VN and Ca_V_1.3_S_-VC and found that expression of these channels increased the number of Ca_V_1.3 channel clusters in the cells, but not the size of the clusters (Figure 10—figure supplemental 1). These results indicate that the spontaneous fusion of Ca_V_1.3_S_ channels was not a consequence of their being over-expressed in the plasma membranes of the hippocampal neurons.

A testable prediction of these observations is that Ca_V_1.3_S_ channel fusion—forced coupling—should augment the inward *I_Ca_* and consequently increase neural excitability and firing rate. Figure 10B shows the spontaneous action potentials recorded from a neuron transfected with Ca_V_1.3_S_-CIBN and Ca_V_1.3_S_-CRY2, before (right trace) and after (center trace) exposure to 488 nm light. Forced coupling of Ca_V_1.3_S_-CIBN and Ca_V_1.3_S_-CRY2 channel pairs increased the average firing rate by about 40% (p<0.01, Figure 10C). 488 nm illumination produced no effect on firing rate in control hippocampal neurons transfected only with Ca_V_1.3_S_-CIBN channels (0.90 ± 0.04, N = 4). Adding the L-type calcium channel blocker nifedipine 10 μM to the bathing solution abolished all AP activity after light-induced fusion of Ca_V_1.3_S_-CIBN and Ca_V_1.3_S_-CRY2 (Figure 10B, left trace and 10C). Together, these results highlight the crucial role that Ca_V_1.3_S_ channels play in regulating the action potential firing in hippocampal neurons.

## Discussion

We have found that Ca_V_1.3 channels in the plasma membrane of hippocampal neurons are arranged in clusters containing multiple (~8) channels. This clustering has important physiological consequences for the short-splice variant of the channel (Ca_V_1.3_S_), enabling proximal channels to engage in cooperative gating, generating more persistent and greater Ca^2+^ influx. Functional coupling of Ca_V_1.3_S_ channels is promoted by intracellular Ca^2+^ and involves physical interactions via channel C-termini mediated by physical interactions between Ca^2+^-CaM and the pre-IQ domain. We propose that Ca^2+^-driven Ca_V_1.3_S_ channel coupling constitutes a novel feed-forward mechanism for the activation of these channels during membrane depolarization and provides an apparatus for Ca^2+^-dependent facilitation of inward L-type Ca^2+^ currents. Further, these findings challenge a fundamental tenet of the classic Hodgkin-Huxley model of ion channel gating: that voltage-gated channels open and close independently.

**Figure 11.**
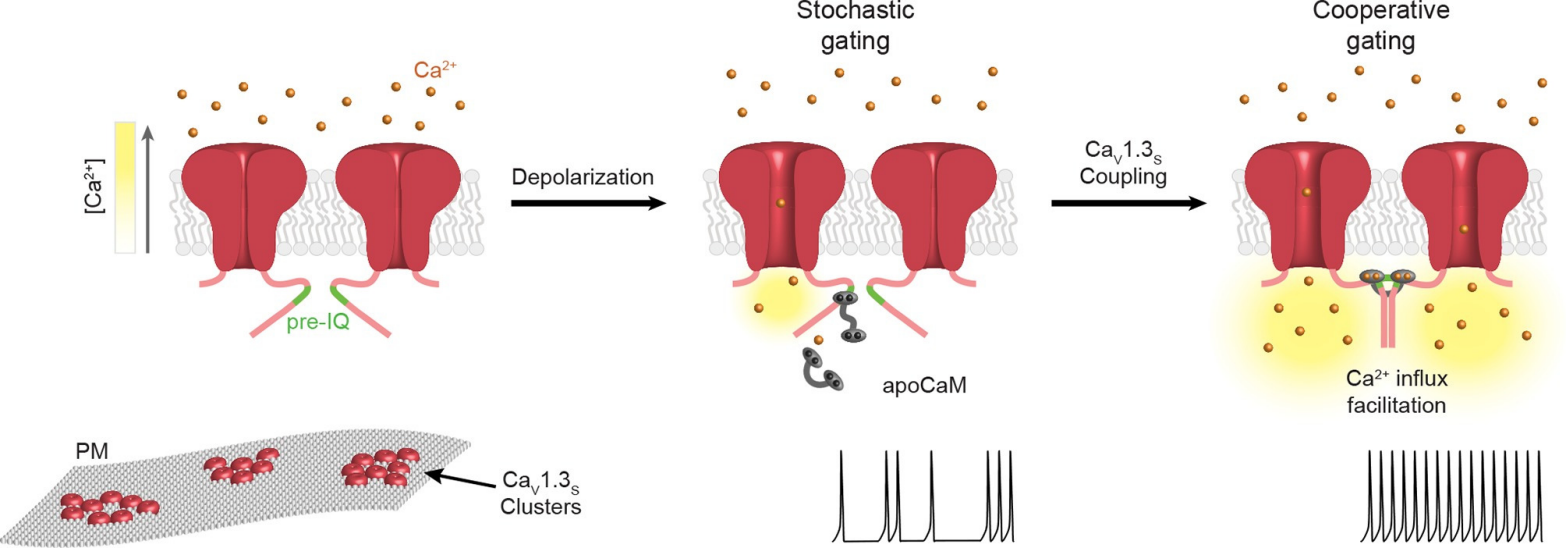
Proposed mechanism for Ca^2+^-induced functional Ca_V_1.3_S_ coupling in hippocampal neurons. Ca_V_1.3_S_ channels are organized in clusters in the plasma membrane (SM) of hippocampal neurons. At hyperpolarized potentials (e.g., -80 mV), where [Ca^2+^]_i_ and P_o_ of Ca_V_1.3_S_ channels is very low, the number of coupled channels is very low. Membrane depolarization increases the probability of stochastic (i.e., uncoupled) openings of Ca_V_1.3_S_ channels. Ca^2+^ flow through these channels creates a local increase in [Ca^2+^]_i_ (yellow gradient). This Ca^2+^ binds to apoCaM, which can be tethered to the pre-IQ domain or soluble in the cytoplasm. Once CaM is activated it promotes channel-channel interaction at the pre-IQ domain. Upon association, the activity of adjoined channels increases, entering into a cooperative gating mode that facilitates Ca^2+^ influx and underlies the ‘depolarizing drive’ that sustain repetitive firing. **DOI:** http://dx.doi.org/10.7554/eLife.15744.020

Our data suggest that Ca_V_1.3_S_ channel coupling is critically dependent on intracellular Ca^2+^ and CaM. During membrane depolarization, Ca_V_1.3_S_ channels open. Ca^2+^ flowing through these channels creates a local increase in [Ca^2+^]_i_ — a Ca_V_1.3 sparklet — near the mouth and C-terminus of the channel where the Ca^2+^-binding protein CaM resides. Upon binding Ca^2+^, CaM associates with the pre-IQ domain of the channels, enabling the formation of Ca_V_1.3_S_-Ca_V_1.3_S_ ‘couplets’. The observation of pre-IQ dimers of the structurally similar Ca_V_1.2 channel undergoing coiled-coil interactions in vitro (Fallon et al., 2009) and functionally couples to neighboring channels (Dixon et al., 2015) gives credence to this model. An interesting question suggested by this model is which CaM pool is involved in Ca_V_1.3_S_ channel coupling? The observation that CaM_1234 _and MLCKp prevent Ca_V_1.3_S_ coupling would suggest that soluble as well as apo-CaM pre-associated with the channel could be involved in inducing physical channel-to-channel interactions. Because physically coupled Ca_V_1.3_S_ channels exhibit higher open probabilities, the overall activity of Ca_V_1.3_S_ channels within a cluster would then depend on the number of channels forming dimers or the probability of the formation of higher order oligomers. A schematic summary of our model is presented in Figure 11.

It is important to note that Minor and colleagues reported the formation of symmetric dimers of pre-IQ domains bridged by two CaM molecules (Kim et al., 2010). However, they failed to obtain any clustering when the channels were expressed in *Xenopus* oocytes. The cause for this apparent lack of clustering of Ca_V_1.2 channels in the frog egg are unclear, but suggest that clustering and functional coupling may be features of Ca_V_1 channels expressed only in mammalian cells, as we have shown here for Ca_V_1.3_S_ channels.

Our results suggest that while close proximity is necessary, it is certainly not sufficient to allow channel interactions. Super-resolution imaging shows that Ca_V_1.3_L_ and Ca_V_1.3_S_ channels form clusters of similar size along the surface membrane, but Ca_V_1.3_L_ channels, unlike Ca_V_1.3_S_ channels, do not undergo physical and functional coupling. Our experimental results with Ca_V_1.3_L_∆116 channels open the question as to whether there is another regulatory domain inside the long C-terminus, capable of blocking the interaction of CaM with the pre-IQ domain. These results also suggest that the mechanism allowing neighboring Ca_V_1.3_S_ channels to interact seems to be structural rather than organizational.

Although Ca_V_1.3 channels are generally classified as high-voltage activated channels, their lower activation range allows them to generate subthreshold depolarizations that support repetitive firing (Olson et al., 2005). This is in particular true for the short variants of Ca_V_1.3, which are more voltage-sensitive than Ca_V_1.3_L_ channels (Bock et al., 2011; Tan et al., 2011). Our results reveal a new and striking characteristic of Ca_V_1.3_S_ channels: they can coordinate their openings through a physical interaction to facilitate Ca^2+^ entry at low membrane potentials. In addition, the fact that Ca_V_1.3 channels are clustered in dendritic spines in these neurons (Gao et al., 2006; Jenkins et al., 2010) raises the possibility that their coordinated gating and boosting of Ca^2+^ entry may have a significant effect on synaptic plasticity.

Subthreshold activation of Ca_V_1.3 channels is also a key component of pacemaking and oscillatory behavior in neurons in the brain, such as dopaminergic neurons in the substantia nigra, the principal neurons affected in Parkinson’s disease (Guzman et al., 2009; Puopolo et al., 2007). In this context, it is tempting to speculate that alterations in the cooperative gating of Ca_V_1.3_S_ might contribute to the Ca^2+^ excitotoxicity observed in multiple pathological conditions, including Parkinson’s neurodegeneration. Future studies should examine the extent to which the cooperative gating of Ca_V_1.3_S_ channels can affect the Ca^2+^ load in neurons.

In summary, our data indicate that Ca^2+^-driven physical interactions among clustered Ca_V_1.3_S_ channels lead to cooperative gating of these channels and the enhanced Ca^2+^ influx that underlies the self-sustained firing of hippocampal neurons. It is anticipated that future studies may reveal that cooperative Ca_V_1.3_S_ channel gating plays an important role in pathological conditions such as Parkinson’s disease, spasticity, and memory loss, as well as in physiological functions as diverse as hearing and the modulation of heart rate, where Ca_V_1.3 channels play a key role.

Finally, our findings point to a novel, general mechanism for the dynamic modulation of ionic currents through L-type Ca_V_1.3_S_ and Ca_V_1.2 channels. For example, in SA node cells, Ca^2+^ entry through Ca_V_1.3 and Ca_V_1.2 channels is a key feature in the generation of pacemaker activity (Mangoni et al., 2003; Platzer et al., 2000; Striessnig et al., 2014). In addition, it has been shown in a recent paper that Ca_V_1.3 channels play a critical role in controlling pacemaker activity in SAN cells through the activation of RyR and the triggering of local Ca^2+^ release from the sarcoplasmic reticulum (SR) (Torrente et al., 2016). The present results suggest a potential new mechanism for Ca^2+^-dependent control of pacing in these cells. Accordingly, spontaneous junctional SR Ca^2+^ release events could induce multimerization of nearby Ca_V_1.3_S_, which, once fused, could produce persistent inward Ca^2+^ currents that increase the number of RyR activated, thereby driving SA node cells closer to the threshold for action potential generation.

## Materials and methods

### Plasmid constructs and tsA-201 cell transfection

The pcDNA clones of the rat Ca_V_1.3 isoforms were obtained from Addgene. We used two Addgene Ca_V_1.3_S_ plasmids#26576 and #49333 (Xu and Lipscombe, 2001), the first one containing two single point substitutions, a glycine by a serine at position 244 and an alanine by a valine at position 1104. We found that they encoded channels with similar voltage-dependencies of activation and rate of activation (Figure 5—figure supplement 2A and B). The Ca_V_1.3_S_ channels encoded by these plasmids were also capable of undergoing Ca^2+^-dependent dimerization and functional coupling (Figure 5—figure supplement 2C–F). On the basis of these data, we concluded that the G244S and A1104V substitutions in the plasmid #26576 are functionally silent with respect to voltage dependence, rate of activation and have no effect on the capacity of adjacent Ca_V_1.3_S_ channels to undergo allosteric interactions. A prior study by Lieb et al. also reported no contribution of these mutations to the functional properties of Ca_V_1.3_S_ channels (Lieb et al., 2012),. Both plasmids #26576 and #49333 were used to express and design the functional Ca_V_1.3_S_ constructs used in this study. Ca_V_1.3_L_ was also obtained from Addgene (plasmid #49332, (Xu and Lipscombe, 2001)). Auxiliary subunits Ca_V_β_3_ and Ca_V_α_2_δ_1_ were gift of Dr. Diane Lipscombe’s laboratory; Brown University, Providence, RI). The C-terminus of Ca_V_1.3_S_ andCa_V_1.3_L_ channels were fused to different proteins depending on the experimental approach. For bimolecular fluorescence complementation, they were fused to either VN or VC fragments of the Venus protein (Kodama and Hu, 2010) (Dr. Chang-Deng Hu, Addgene plasmids 27097, 22011); for photobleaching experiments, they were fused to monomeric GFP (mGFP_A206K_), amplified from the pCGFP-EU vector (Kawate and Gouaux, 2006), kindly provided by Dr. Eric Goaux (Oregon Health and Science University, Portland, OR); and for the light induced cryptochrome system, they were fused with either CRY2 or CIBN (generous gifts from Dr. Pietro Di Camilli, Yale University, New Haven, CT). The CaM_1234_ plasmid was a generous gift from Dr. Johannes Hell (University of California, Davis, CA).

The tsA-201 cell line, used for heterologous expression of the constructs listed above, was maintained in Dulbecco’s modified Eagle medium supplemented with 10% fetal bovine serum and 1% penicillin/streptomycin antibiotic solution. Cells were transiently transfected using jetPEI transfection reagent (Polyplus Transfection, New York, NY) and plated onto 25-mm coverslips (0.13–0.17-mm thick). Successfully transfected cells were identified on the basis of turbo red fluorescent protein (tRFP) fluorescence. Imaging and electrophysiology experiments were performed within 48 hr of transfection.

### Hippocampal neuron culture and transfection

Hippocampal neurons were prepared from newborn (P1) Sprague-Dawley rats in accordance with University of Washington (UW) guidelines. Animals were decapitated and their tissue harvested according to a protocol approved by the UW Institutional Animal Care and Use Committee (IACUC). The hippocampi of six rat pups were dissected and cut into small pieces in cold dissection medium consisting of 12 mM MgSO_4_ and 0.3% bovine serum albumen (BSA) in Hank’s balanced salt solution (HBSS). The pieces were incubated for 30 min at 37ºC in dissection medium containing 25 U/ml papain. The digested tissue was washed with warm Neuronal medium consisting of Minimal Essential Medium (MEM) supplemented with 10% horse serum, 2% B27, 25 mM HEPES, 20 mM glucose, 2 mM GlutaMAX, 1 mM sodium pyruvate, and 1% penicillin/streptomycin antibiotic solution. The tissue was then gently homogenized in fresh Neuronal medium using a long Pasteur pipette. Neurons were plated on poly-D-lysine-coated coverslips (0.2 mg/ml for 2 hr) at a density of 2 x 10^5^ cells/coverslip. After incubating neurons at 35ºC for 24 hr, unattached cells were removed by replacing the medium with fresh Neuronal medium. Every 5 day, one-third of the medium was replaced with fresh Neuronal medium supplemented with the anti-mitotic agents fluorodeoxyuridine (20 μM) and uridine (50 μM).

After 14 d in culture, rat hippocampal neurons were transfected using 2.4 μg DNA, 4.8 μl of Lipofectamine LTX and 4.8 μl PLUS reagent (Life Technologies, Grand Island, NY) in a final volume of 1 ml of a 1:1 mixture of Neuronal medium and Opti-MEM. After 1 hr of incubation, the medium was replaced with fresh Neuronal medium. Experiments were performed within 48 hr of transfection. Successful transfection was corroborated by detection of tRFP.

### Electrophysiology

Ca^2+^ currents were recorded using the whole-cell configuration of the patch-clamp technique in voltage-clamp mode. Currents were sampled at a frequency of 10 kHz and low-pass filtered at 2 kHz using an Axopatch 200B amplifier. During the experiments, tsA-201 cells were superfused with a solution containing 5 mM CsCl, 10 mM HEPES, 10 mM glucose, 140 mM N-methyl-D-glucamine, 1 mM MgCl_2_ and 2 mM CaCl_2_ or 2 mM BaCl_2_, depending on the experiment. pH was adjusted to 7.4 with HCl. For the experiments using 20 mM CaCl_2_, the osmolarity was adjusted by decreasing the concentration of NMDG to 113 mM. Borosilicate patch pipettes with resistances of 3–6 MΩ were filled with an internal solution containing 87 mM cesium aspartate (CsAsp), 20 mM CsCl, 1 mM MgCl_2_, 10 mM HEPES, 10 mM EGTA and 5 mM MgATP, adjusted to pH 7.2 with CsOH. A voltage offset of 10 mV, attributable to the liquid junction potential of these solutions, was corrected offline. Current–voltage relationships were obtained by subjecting cells to a series of 300-ms depolarizing pulses from a holding potential of -80 mV to test potentials ranging from -70 to +50 mV. The voltage dependence of channel activation (G/G_max_) was obtained from the resultant currents by converting them to conductances using the equation, G = *I_Ca_*/(test pulse potential – reversal potential of *I_Ca_*); normalized G/G_max_ was plotted as a function of test potential. We found that the reversal potential for Ca_V_1.3_L_ (+51 ± 4 mV) and Ca_V_1.3_S_ (+54 ± 3 mV) currents in the presence of 2 mM Ca^2+^ were not significantly different (p=0.49). The same was true with 2 mM Ba^2+^ in the bath (Ca_V_1.3_L_ = +60 ± 5 mV; Ca_V_1.3_S_ = +65 ± 2 mV; p=0.31). Thus, while E_rev_ is similar in Ca_V_1.3_L_ and Ca_V_1.3_S_ channels with the same permeating ion, the reversal potential of Ca^2+^ and Ba^2+^ currents through these channels differ. In our patch clamp experiments in which the external solution was switched from Ba^2+^ to Ca^2+^, 2-min intervals were inserted between the onset of the whole cell configuration to the first pulse and again after switching the external solution, to rule out any effect of the run-up of the *I_Ca_* (Tiaho et al., 1993).

Single-channel currents (*i_Ca_*) were recorded from tsA-201 using the cell-attached configuration. Cells were superfused with a high K^+^ solution to fix the membrane potential at ∼0 mV. The bathing solution had the following composition: 145 mM KCl, 2 mM MgCl_2_, 0.1 mM CaCl_2_, 10 mM HEPES and 10 mM glucose; pH was adjusted to 7.3 with KOH. Pipettes were filled with a solution containing 10 mM HEPES and either 20 mM CaCl_2_ or 20 mM BaCl_2_; pH was adjusted to 7.2 with CsOH. The dihydropyridine agonist BayK-8644 (500 nM) was included in the pipette solution to promote longer channel open times. A voltage-step protocol from a holding potential of −80 mV to a depolarized potential of −30 mV was used to elicit currents. The single-channel event-detection algorithm of pClamp 10.2 was used to measure single-channel opening amplitudes. We generated all-points histograms from our cell-attached patch-clamp recordings. These histograms were fit using Prism 5.0a software (GraphPad software Inc. La Jolla, CA) with a multi-Gaussian function that included a quantal (*q*; i.e., elementary current) parameter using the following equation:

$$N=\sum_{j=1}^{n}\;a_{j}\times e^{\left[-\frac{i_{Ca}-jq^{2}}{2jb}\right]},$$

where *N* is the number of events, *a* and *b* are constants, *i_Ca_* is the amplitude of the current measured and *q* is the quantal elementary current of the channel.

Spontaneous discharge of cultured hippocampal neurons was recorded in current-clamp mode using the perforated-patch configuration. Neurons were superfused with a solution containing 140 mM NaCl, 5 mM KCl, 10 mM HEPES, 10 mM glucose, 1 mM MgCl_2_, 2 mM CaCl_2_ and 1 mM Na-pyruvate, adjusted to pH 7.4 with NaOH. Borosilicate patch pipettes with resistances of 3–6 MΩ were filled with an internal solution containing 5 mM NaCl, 140 mM KCl, 15 mM HEPES and 7 mM MgATP, adjusted to pH 7.2 with KOH; 60 μM amphotericin B was added to the solution before starting the recording. Series resistances lower than 30 MΩ were obtained within 5 min of seal formation. The sampling frequency was 10 kHz filtered at 2 kHz.

### Immunofluorescence and super-resolution microscopy

For immunostaining Ca_V_1.3 in tsA-201 cells or hippocampal neurons, cells were fixed by incubating in phosphate-buffered saline (PBS) containing 3% paraformaldehyde and 0.1% glutaraldehyde for 15 min. After washing with PBS, cells were incubated with 50 mM glycine at 4ºC for 10 min (aldehyde reduction), washed again with PBS, and blocked by incubating with 20% SEA BLOCK (Thermo Scientific) and 0.25% v/v Triton X-100 in PBS (blocking buffer) for 1 hr. The cells were incubated overnight at 4ºC with primary antibodies recognizing the residues 809 to 825 located at the intracellular II-III loop of the Ca_V_1.3 channel (DNKVTIDDYQEEAEDKD, rabbit; provided by Drs. William Catterall and Ruth Westenbroek) and the neuronal marker MAP2 (mouse; Abcam), diluted in blocking buffer to a concentration of 10 μg/ml. Cells were then washed with PBS, incubated for 1 hr with Alexa Fluor 647-conjugated donkey anti-rabbit (2 µg/ml; Molecular Probes) and Alexa Fluor 488-conjugated chicken anti-mouse (2 µg/ml; Molecular Probes) secondary antibodies, and washed again with PBS. Our antibody was designed to bind to the intracellular loop linking the 2^nd^ and 3^rd^ membrane domains of Ca_V_1.3. It cannot distinguish between Ca_V_1.3_S_ and Ca_V_1.3_L_.

For super-resolution microscopy, coverslips were mounted on microscope slides with a round cavity using MEA-GLOX imaging buffer (NeoLab Migge Laborbedarf-Vertriebs GmbH, Germany) and sealed with Twinsil (Picodent, Germany). The imaging buffer contained 10 mM MEA, 0.56 mg/ml glucose oxidase, 34 μg/ml catalase, and 10% w/v glucose in TN buffer (50 mM Tris-HCl pH 8, 10 mM NaCl).

A super resolution ground-state depletion system (SR-GSD, Leica) based on stochastic single-molecule localization was used to generate super-resolution images of Ca_V_1.3 in hippocampal neurons and tsA-201 cells. The Leica SR-GSD system was equipped with high-power lasers (488 nm, 1.4 kW/cm^2^; 532 nm, 2.1 kW/cm^2^; 642 nm, 2.1 kW/cm^2^) and an additional 30 mW, 405 nm laser. Images were obtained using a 160× HCX Plan-Apochromat (NA 1.43) oil-immersion lens and an EMCCD camera (iXon3 897; Andor Technology). For all experiments, the camera was running in frame-transfer mode at a frame rate of 100 Hz (10 ms exposure time). Fluorescence was detected through Leica high-power TIRF filter cubes (488 HP-T, 532 HP-T, 642 HP-T) with emission band-pass filters of 505–605 nm, 550–650 nm, and 660–760 nm.

Super-resolution localization images of Ca_V_1.3 channel distribution were reconstructed using the coordinates of centroids obtained by fitting single-molecule fluorescence signals with a 2D Gaussian function using LASAF software (Leica). A total of 50,000–100,000 images were used to construct the images. The localization accuracy of the system is limited by the statistical noise of photon counting. Thus, assuming the point-spread functions are Gaussian, the precision of localization is proportional to DLR/√N, where DLR is the diffraction-limited resolution of a fluorophore and N is the average number of detected photons per switching event (Dempsey et al., 2011; Folling et al., 2008). Accordingly, we estimated a lateral localization accuracy of 16 nm for Alexa 647 (~1900 detected photons per switching cycle). Ca_V_1.3 cluster size was determined using binary masks of the images in ImageJ software (NIH).

### Step-wise photobleaching

The number of Ca_V_1.3_S_ channels in clusters along the surface membrane was estimated using a single-molecule bleaching approach similar to that described by Ulbrich and Isacoff (2007). Briefly, TIRF images of tsA-201 or hippocampal neurons expressing Ca_V_1.3_S_ channels fused to the monomeric GFP were acquired using our Leica GSD microscope in TIRF mode. Cells were fixed with 4% paraformaldehyde for 10 min prior to the acquisition. Images were acquired using an oil immersion 160x objective (NA 1.43) and an Andor iXON EMCCD camera. GFP was excited with a 488 nm laser and image stacks of 2000 frames were acquired at 30 Hz. During analysis, the first 5 images of the stack were averaged and a rolling-ball background subtraction was applied using ImageJ (NIH). This image was then low-pass filtered with a 2 pixel cut-off and high-pass filtered with a 5 pixel cut-off. Thresholding was then applied to identify connected regions of pixels that were above threshold. The ImageJ plugin ‘Time Series Analyzer v2.0’ was used to select 4x4 pixel ROIs, centered on the peak pixel in each spot. These ROIs were used to plot Z-axis intensity profiles (where z is time) of the entire image stack to manually detect the bleaching steps.

### Split Venus bi-molecular fluorescence complementation

The spontaneous interaction between the C-terminus of Ca_V_1.3_S_ channels was assessed using the split Venus system (Shyu et al., 2006). In this approach, Ca_V_1.3_S_ channels were fused to either the VN fragments (N_1–154_) or the VC fragment (C_155–238_) of the Venus fluorescent protein. The Venus protein emits fluorescence only when the two fragments are close enough to interact and reconstitute the whole protein, providing a measure of the proximity between the C-terminus of the Ca_V_1.3_S_ channels. Ca_V_1.3_S_ -VN and/or Ca_V_1.3_S_ -VC constructs were expressed at a 1:1 ratio in hippocampal neurons; tRFP fluorescence was used as an indicator of successful transfection. Confocal images were acquired with a Fluoview FV1000 microscope (Olympus, Center Valley, PA) equipped with a UPlanS-Apochromat 60× (NA 1.2) water-immersion objective. The Venus protein was excited using a 488 nm laser line. The calcium dependence of Ca_V_1.3 spontaneous interactions was studied in tsA-201 cells expressing Ca_V_1.3_S_ -VC alone or Ca_V_1.3_S_ -VN and Ca_V_1.3_S_ -VC in a 1:1 ratio. Using the whole-cell configuration of the patch-clamp technique, cells were depolarized from a holding voltage of -80 mV to test potentials ranging from -60 to +60 mV, administered as 9-s pulses. Maturation of newly reconstituted Venus protein takes some time, hence the long depolarizing pulse (Nagai et al., 2002). Images were acquired at a frequency of 100 Hz during each depolarizing pulse using a through-the-lens TIRF microscope built around an inverted microscope (IX-70; Olympus) equipped with a Plan-Apochromat (60×; NA 1.49) oil-immersion lens (Olympus) and an electron-multiplying charge-coupled device (EMCCD) camera (iXON; Andor Technology, UK). The last 10 images of each stack were averaged, and total fluorescence was quantified using ImageJ software (NIH). The images were pseudo-colored using the ‘red hot’ lookup table in ImageJ. F_0_ was calculated by dividing the total fluorescence for each voltage by the initial fluorescence at -80 mV. The change in F/F_0_ was plotted against voltage and compared to G/G_max_ curves constructed as described in the electrophysiology section. The Ca^2+^ dependence of Venus reconstitution was tested in cells bathed in an external solution containing 20 mM Ca^2+^ or 2 mM Ba^2+^.

### Ca_V_1.3_S_ sparklet imaging and analysis

Ca_V_1.3_S_ sparklets were recorded using the TIRF microscopy system described above. [Ca^2+^]_i_ was monitored by adding the Ca^2+^ indicator Rhod-2 (200 µM) to the pipette solution and exciting with a 568 nm laser. The much slower Ca^2+^ buffer EGTA (10 mM) was included with the relatively fast Ca^2+^ indicator, Rhod-2, to restrict Ca^2+^ signals to the vicinity of the Ca^2+^ entry source. Sparklets were detected in tsA-201 cells expressing Ca_V_1.3_S_ channels. The driving force for Ca^2+^ entry was increased by holding the membrane potential at -80 mV using the whole-cell configuration of the patch-clamp technique.

TIRF images were acquired at a frequency of 100 Hz using TILL Image software. Sparklets were detected and analyzed using custom software written in MATLAB (Source code 1). Fluorescence intensity values were converted to nanomolar units as described previously (Navedo et al., 2007). Event amplitude histograms were generated from [Ca^2+^]_i_ records and fitted with a multicomponent Gaussian function. We determined the activity of sparklets by calculating the *nP_s_* of each sparklet site, where *n* is the number of quantal levels and *P_s_* is the probability that a quantal sparklet event is active. A detailed description of this analysis can be found in Navedo et al. (Navedo et al., 2006).

In split Venus experiments, sparklets were acquired at -80 mV before and after a depolarizing protocol from -60 to +60 mV with 9-s pulses. Sparklet images were always acquired in a solution containing 20 mM Ca^2+^, whereas depolarization protocols were run in 20 mM Ca^2+^ or 2 mM Ba^2+^ to compare the effect of Ca^2+^-dependent Ca_V_1.3_S_ dimerization on sparklet activity.

The degree of coupling between Ca_V_1.3 Ca^2+^ sparklet sites was assessed by further analyzing sparklet recordings using a binary coupled Markov chain model, as first described by Chung and Kennedy (1996) and previously employed by our group (Navedo et al., 2010; Cheng et al., 2011; Dixon et al., 2012). The custom program (Source code 2), written in the MATLAB language, assigns a coupling-coefficient (κ) to each record, where κ can range from 0 (purely independently gating channels) to 1 (fully coupled channels). Elementary event amplitudes were set at 38 nM.

### Cryptochrome light-induced Ca_V_1.3 dimerization

Light-induced dimerization of Ca_V_1.3 channels was accomplished by fusing Ca_V_1.3 channels with the optogenetic light-induced dimerization system based on CRY2 and CIB1 proteins of *Arabidopsis thaliana* (Kennedy et al., 2010). Upon blue-light illumination (488 nm), CRY2 absorbs a photon, causing a conformational change in one of its domains that promotes binding to the N-terminal region of CIB1 (CIBN). Ca_V_1.3-CIBN and/or Ca_V_1.3-CRY2 constructs were expressed in a 1:1 ratio in hippocampal neurons. Forty-eight hours after transfection, spontaneous action potential firing was recorded in current-clamp mode. One minute after initiating recordings, neurons were exposed to a 30-s blue light pulse to induce dimerization, and the changes in the firing pattern were measured. In tsA-201 cells, Ca^2+^ currents were recorded before and after light illumination in response to a 20-ms depolarizing pulse at +10 mV from a holding potential of -80 mV. Experiments were performed on a Nikon (Eclipse TE2000-S) Swept Field confocal system controlled with Elements software and equipped with a 488 nm laser line and a Plan Apo 60× 1.45 N.A. oil-immersion objective.

### Data analysis

Data were collected from at least five independent experiments in each series. The data included in this paper were normally distributed. Accordingly, parametric statistics were performed and mean ± SEM are used to provide a description of the data set. Student’s t-test was used to test for statistical significance using Prism 5.0 a software (GraphPad software Inc. La Jolla, CA). We decided, *a priori*, that p values <0.05 were indicative a statistical significance difference between or among data groups. The number of cells used for each experiment and *p* values are detailed in each figure legend. Paired t- tests were used to test for statistical significance of paired observations. Comparisons between three or more conditions were made by one-way ANOVA test using Prism software.

## Additional files

Source code 1. Custom software for Ca2+ sparklet detection and analysis written in MATLAB. **DOI:** http://dx.doi.org/10.7554/eLife.15744.021

Source code 2. Binary coupled Markov chain model. **DOI:** http://dx.doi.org/10.7554/eLife.15744.022
